# De Novo Formation of Insulin-Producing “Neo-β Cell Islets” from Intestinal Crypts

**DOI:** 10.1016/j.celrep.2014.02.013

**Published:** 2014-03-06

**Authors:** Yi-Ju Chen, Stacy R. Finkbeiner, Daniel Weinblatt, Matthew J. Emmett, Feven Tameire, Maryam Yousefi, Chenghua Yang, Rene Maehr, Qiao Zhou, Ruth Shemer, Yuval Dor, Changhong Li, Jason R. Spence, Ben Z. Stanger

**Affiliations:** 1 Gastroenterology Division, Department of Medicine, University of Pennsylvania, Philadelphia, PA 19104, USA; 2 Department of Cell and Developmental Biology, University of Pennsylvania, Philadelphia, PA 19104, USA; 3 Abramson Family Cancer Research Institute, University of Pennsylvania, Philadelphia, PA 19104, USA; 4 Department of Radiation Oncology, University of Pennsylvania, Philadelphia, PA 19104, USA; 5 Department of Pediatrics, Perelman School of Medicine, University of Pennsylvania, Philadelphia, PA 19104, USA; 6 Program in Molecular Medicine, University of Massachusetts Medical School, University of Massachusetts, Worcester, MA 01655, USA; 7 Department of Stem Cell and Regenerative Biology, Harvard University, Cambridge, MA 02138, USA; 8 Department of Developmental Biology and Cancer Research, The Hebrew University-Hadassah Medical School, Jerusalem 91120, Israel; 9 Gastroenterology Division, Department of Internal Medicine, University of Michigan, Ann Arbor, MI 48109, USA; 10 Department of Cell and Developmental Biology, University of Michigan, Ann Arbor, MI 48109, USA; 11 Center for Organogenesis, Department of Medicine, University of Michigan, Ann Arbor, MI 48109, USA

## Abstract

The ability to interconvert terminally differentiated cells could serve as a powerful tool for cell-based treatment of degenerative diseases, including diabetes mellitus. To determine which, if any, adult tissues are competent to activate an islet β cell program, we performed an in vivo screen by expressing three β cell “reprogramming factors” in a wide spectrum of tissues. We report that transient intestinal expression of these factors—Pdx1, MafA, and Ngn3 (PMN)—promotes rapid conversion of intestinal crypt cells into endocrine cells, which coalesce into “neoislets” below the crypt base. Neoislet cells express insulin and show ultrastructural features of β cells. Importantly, intestinal neoislets are glucose-responsive and able to ameliorate hyperglycemia in diabetic mice. Moreover, PMN expression in human intestinal “organoids” stimulates the conversion of intestinal epithelial cells into β-like cells. Our results thus demonstrate that the intestine is an accessible and abundant source of functional insulin-producing cells.

## INTRODUCTION

Type 1 and type 2 diabetes are associated with either destruction or dysfunction of pancreatic β cells. The ability of cadaveric islet transplantation to restore euglycemia in patients with severe type 1 diabetes—the “Edmonton protocol”—has fueled efforts to create a reproducible and immune-compatible source for new β cells (Shapiro et al., 2000). Such efforts, particularly those utilizing human embryonic stem cells (ESCs) or induced pluripotent cells (iPSCs), have made some headway (Cheng et al., 2012; Jiang et al., 2007; Kroon et al., 2008). Nevertheless, there have been conflicting reports, and a number of significant technical issues remain (D'Amour et al., 2006; Kroon et al., 2008).

The discovery that ectopic expression of four transcription factors can reprogram a terminally differentiated cell to pluripotency has reinvigorated the field of somatic cell transdifferentiation (Takahashi and Yamanaka, 2006). In the pancreas, adenovirus-mediated introduction of three transcription factors involved in normal islet development—Pdx1 (P), MafA (M), and Ngn3 (N)—can induce acinar cells to become insulin-producing cells in an immune-deficient mouse (Zhou et al., 2008), raising the prospect of using differentiated pancreatic cells as a source for new β cells. These findings have been partially reproduced in cultured cells, although the cells that arise following “reprogramming” in vitro lack many of the features of functional β cells (Akinci et al., 2012; Hickey et al., 2013).

Given recent studies indicating that adult cells retain a high degree of cellular plasticity (Ieda et al., 2010; Kajimura et al., 2009; Takahashi and Yamanaka, 2006; Vierbuchen et al., 2010; Xie et al., 2004; Yanger et al., 2013; Zhou et al., 2008), we reasoned that misexpression of the PMN transcription factors in a wide variety of tissues might permit identification of other cell types that can undergo conversion to a β or β-like state in vivo. In the current study, we ectopically expressed the PMN factors in an immune-competent mouse to screen for such tissues.

## RESULTS

### Widespread Expression of PMN Factors In Vivo

**Figure 1:**
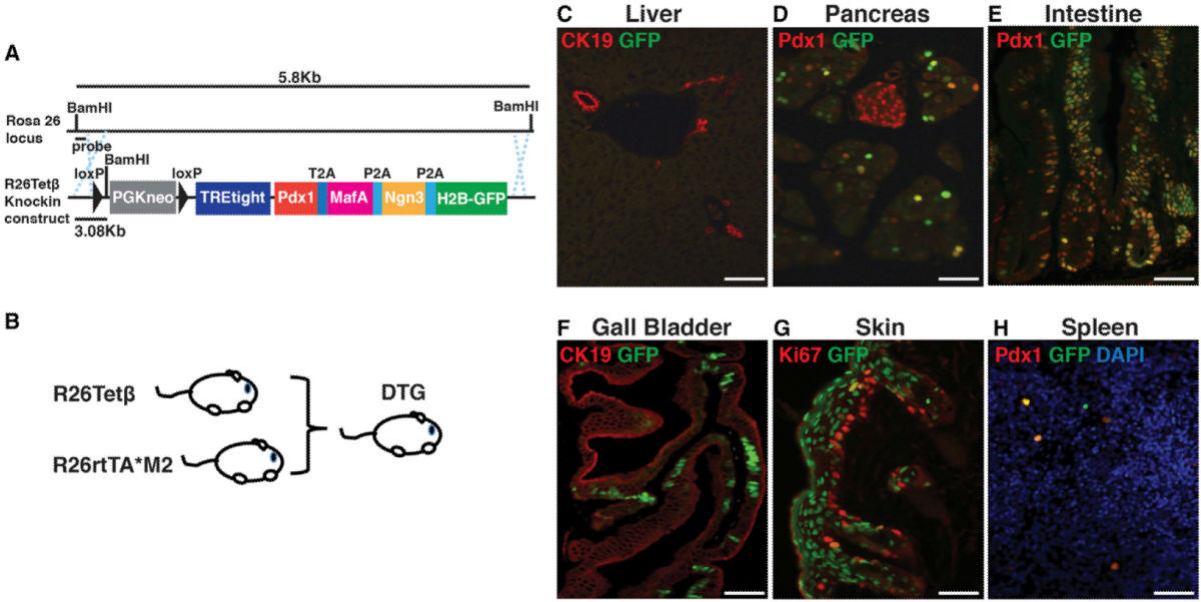
An In Vivo Screen for Tissues Competent to Initiate Insulin Transcription (A) Schematic representation of transgenes used to generate Dox-inducible Tetß mice. A cassette containing *Pdx1*, *MafA*, *Ngn3*, and *H2B-GFP* cDNAs linked by 2A peptide sequences (T2A, P2A) under the tetracycline-responsive promoter (TRE-tight) was targeted into the *Rosa26* locus, resulting in the R26Tetß-targeting construct. (B) Schematic showing breeding of R26Tetß and R26rtTA*M2 mice to generate double-transgenic (DTG) mice. Mice bearing either the R26Tetß or R26rtTA*M2 transgene served as controls. (C–H) GFP induction in DTG tissues after 4 days of Dox treatment. Immunofluorescence images showing GFP and costaining with the indicated markers. GFP was detected by direct epifluorescence. Note the absence of GFP in the liver (C) and pancreatic islets (D). Coexpression of GFP and Pdx1 is shown in pancreatic acinar cells (D), intestinal epithelial cells (E), and spleen cells (H). The scale bars represent 25 μm. See also Figure S1.

We combined a 2A self-cleaving peptide-based strategy (Szymczak et al., 2004) with an inducible doxycycline-dependent expression (Tet-On) system to investigate the effect of ectopic PMN expression in vivo. Initially, the PMN factors and an H2B-Cherry reporter were connected by 2A peptide sequences and cloned in-frame into the FUW lentiviral backbone to generate *pLenti-Beta* (Figure S1A, top). Expression of all three proteins, as well as the H2B-mCherry reporter, was confirmed in 293T cells and a human hepatocyte cell line (Figures S1A and S1B). We then generated *R26Tet*ß ESCs by replacing H2B-Cherry sequences with an H2B-GFP reporter, cloning the PMN-GFP open reading frame downstream of a tetracycline response element (TREtight) and targeting the resulting *Tet*ß cassette to the *Rosa26* locus of mouse ESCs. Next, we generated *R26Tet*ß mice and crossed them to R26-rtTA*M2 mice (Hochedlinger et al., 2005), permitting widespread doxycycline (Dox)-regulated expression of the PMN factors and the GFP reporter in double transgenic (DTG) animals (Figure 1B). Without Dox treatment, there was no GFP (Figure S1C). Following 3 days of Dox treatment, GFP and PMN factors were detected in the pancreas, intestine, gallbladder, skin, spleen, and bone marrow, but not liver, lung, heart, or kidney (Figures 1C–1H and S1E; data not shown). Within the pancreas, expression was observed in the exocrine compartment, but not in islets (Figures 1D and S1E).

### Robust Induction of Insulin-Secreting Cells in the Intestine

**Figure 2:**
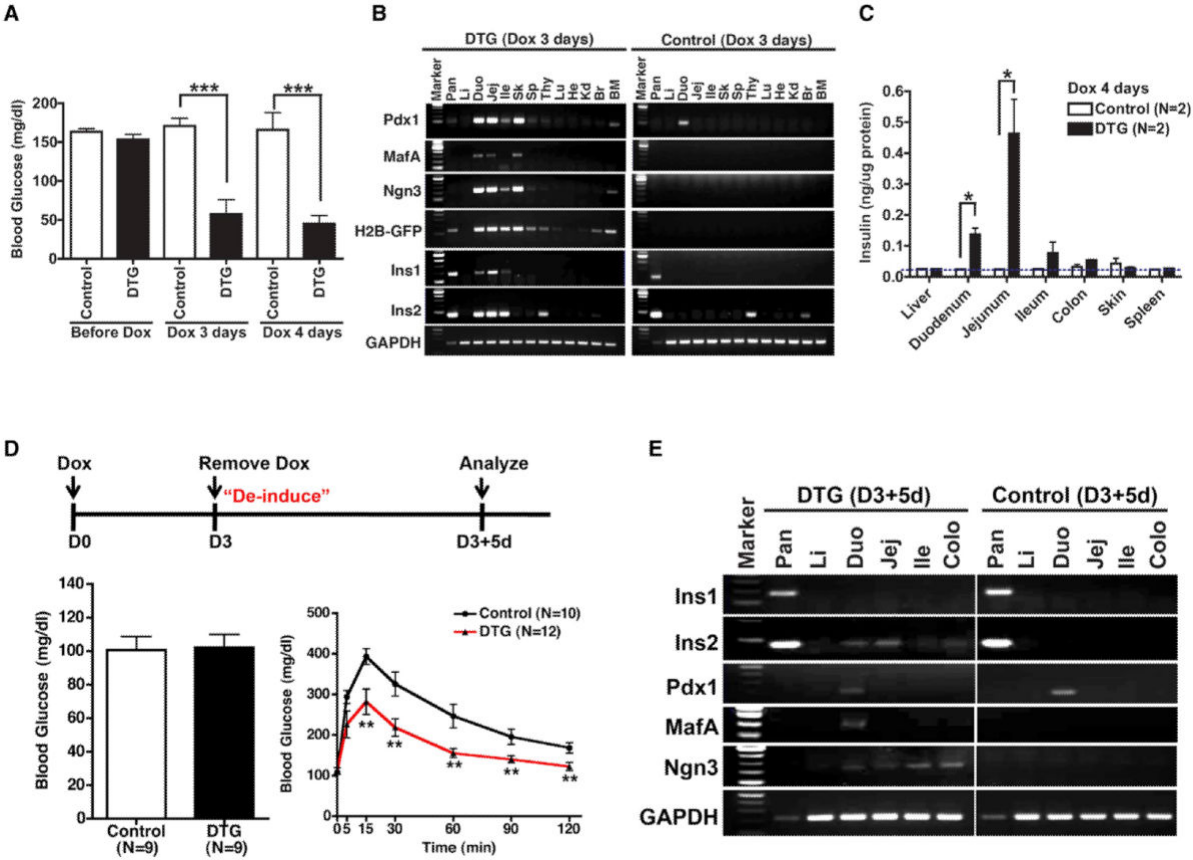
Systemic Effects of Intestinal Insulin following PMN Expression (A) Blood glucose levels of DTG mice following 3 to 4 days Dox treatment (measured after 1 hr fast). ***p < 0.001, Student's t test. (B) RT-PCR analysis in multiple tissues from DTG mice and controls treated with 3 days Dox. GAPDH served as a control for template cDNA. BM, bone marrow; Br, brain; Duo, duodenum; He, heart; Ile, ileum; Jej, jejunum; Kd, kidney; Li, liver; Lu, lung; Pan, pancreas; Sk, skin; Sp, spleen; Thy, thymus. (C) Measurement of insulin protein in various tissues by ELISA. *p < 0.05, Student's t test. (D) Top: Schematic for induction of PMN factors for 3 days with Dox and then “deinduction” for 5 days following Dox removal. Lower left: D3+5d animals have normal fasting blood glucose (17 hr fast). Lower right: D3+5d animals have improved glucose homeostasis following intraperitoneal glucose challenge. **p < 0.01, Student's t test. (E) RT-PCR analysis of *insulin* (Ins1 and Ins2), *Pdx1*, *MafA*, and *Ngn3* transcript abundance in multiple tissues from D3+5d deinduced mice. See also Figure S2.

DTG mice were analyzed for effects on glucose homeostasis and insulin production. After 3 to 4 days of Dox administration, DTG animals exhibited a profound decrease in blood glucose (BG) levels in the fed state (Figure 2A) and overt symptoms of hypoglycemia, including lethargy, seizure, and coma. To determine whether hypoglycemia might be due to ectopic insulin production, we examined RNA from a variety of DTG mouse tissues after 3 days of Dox (D3 DTG) for *insulin* transcripts. Surprisingly, we found transcripts for *Ins1* and *Ins2* in the duodenum, jejunum, and ileum in addition to tissues in which *insulin* is normally transcribed—pancreas, thymus, and brain (Figure 2B). We confirmed ectopic insulin production at the protein level by ELISA, which revealed significantly elevated levels in the duodenum and jejunum, but not in other parts of the intestine or in other tissues (Figure 2C). Notably, there was no increase in pancreatic insulin content at this time point, and staining failed to detect acinar cells that expressed either the endocrine marker ChromograninA (ChroA) or insulin itself. Specifically, we examined 1,360 GFP^+^ acinar cells (out of a total of 5,818 acinar cells counted; n = 2), and none costained for insulin (Figure S1D; data not shown). These results suggest that hypoglycemia in Dox-treated DTG animals results from intestinally derived insulin production.

We then developed a protocol whereby animals were treated with Dox for 3 days and then “deinduced” by removing Dox for 5 days prior to analysis (D3+5d; Figure 2D). Under these conditions, BG levels dropped transiently during Dox treatment and then rose following Dox withdrawal (Figure S2A), resulting in a normal fasting BG in D3+5d DTG animals (Figure 2D). Despite this, such animals still exhibited an improved response to glucose challenge compared to control littermates (Figure 2D). Transcripts for *Ins2* (but not *Ins1*) persisted in the intestines of D3+5d animals, as did transcripts for *Pdx1*, *MafA*, and *Ngn3* (Figure 2E). Consistent with our earlier immunofluorescence (IF) experiments showing a lack of acinar insulin staining, there was no increase in total pancreatic insulin content in D3+5d animals (Figure S2B). Notably, deinduced DTG animals exhibited a trend toward early and enhanced insulin release into the blood following glucose challenge (Figure S2C). Thus, intestinal expression of *Pdx1*, *MafA*, and *Ngn3* drives the formation of ectopic insulin-producing cells that confer an improved response to glucose challenge without fasting hypoglycemia.

### Intestinal Insulin^+^ Cells Are Epithelially Derived and Form “Neoislets”

**Figure 3:**
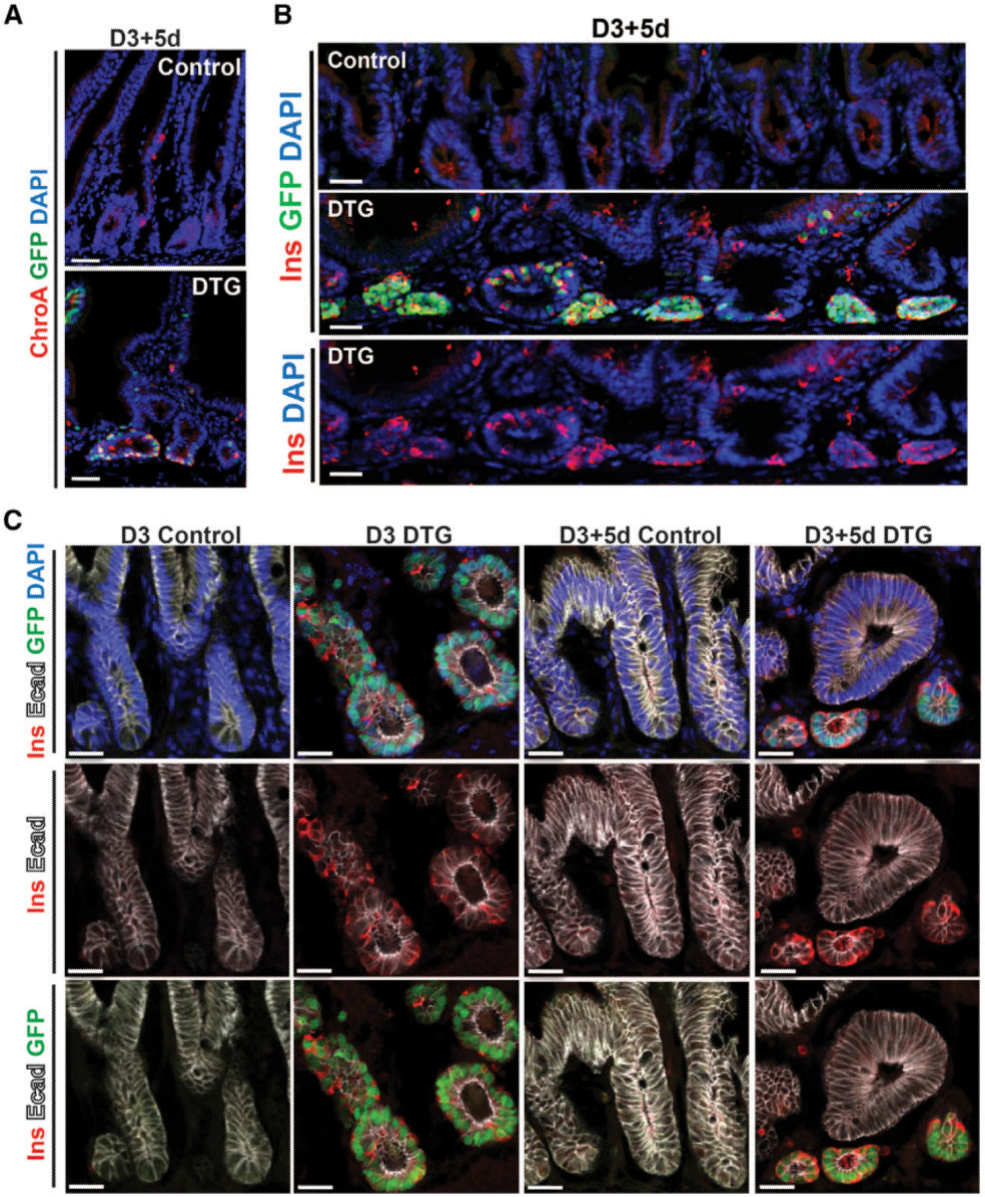
Intestinal Insulin^+^ Cells Are Epithelially Derived and Form Neoislets (A and B) Representative immunofluorescence images of D3+5d DTG intestines stained for ChroA (A) and insulin (B). H2B-GFP marks “label-retaining” (nondividing) cells in the intestine in deinduced D3+5d mice. (C) Representative sections of D3 and D3+5d mice intestine stained for insulin (Ins), E-cadherin (Ecad), and DAPI. The scale bars represent 25 μm. See also Figure S3.

In contrast to control intestines, in which rare ChroA^+^ enteroendocrine cells were scattered in the differentiated regions of the intestinal villi, ChroA^+^ cells were abundant in the undifferentiated crypt regions of D3 DTG intestines, where most cells were also positive for GFP (data not shown). Following deinduction (D3+5d), there were fewer ChroA^+^/GFP^+^ cells, and those that remained persisted as islet-like clusters lying near or below the crypt bases (Figure 3A). 5-ethynyl-2′-deoxyuridine and phosphohistone 3 staining suggested that these cells were nonproliferative (Figure S3A; data not shown). Thus, H2B-GFP retention served as a marker for cells that had previously expressed the PMN factors. We refer to these clusters as neoislets.

We then stained D3 and D3+5d DTG intestines for various pancreatic hormones. As expected, IF for insulin showed no staining in the intestines of control animals (Figure 3B). By contrast, we saw abundant insulin^+^ cells within the intestines of D3 DTG animals. Insulin^+^ cells were concentrated in crypts, with some scattered cells in villi (Figure 3C; data not shown). Although this insulin staining never overlapped with somatostatin staining (data not shown), a small fraction of GFP^+^ cells in D3 DTG mice were either glucagon^+^ (71 out of 3,368 GFP^+^ cells; 2.1%) or glucagon^+^ /insulin^+^ (98 out of 3,368 GFP^+^ cells; 2.9%). Insulin^+^ cells made up 35.7% of the GFP^+^ cells at this stage (1,201 out of 3,368 GFP^+^ cells). After 5 days of deinduction (D3+5d), most insulin^+^ cells were present within the neoislet GFP^+^ clusters, where they comprised 31.6% of the GFP^+^ cells (197 out of 623 GFP^+^ cells; n = 2; Figures 3B and 3C). Interestingly, no glucagon^+^ cells were detected in the neois-lets of D3+5d mice (Figure S3B). Confocal microscopy demonstrated that the epithelial marker E-cadherin (Ecad) was present on the membranes of intestinal GFP^+^/insulin^+^ cells in both D3 and D3+5d mice (Figure 3C).

### Intestinal Insulin^+^ Cells Exhibit Features of Functional β Cells

**Figure 4:**
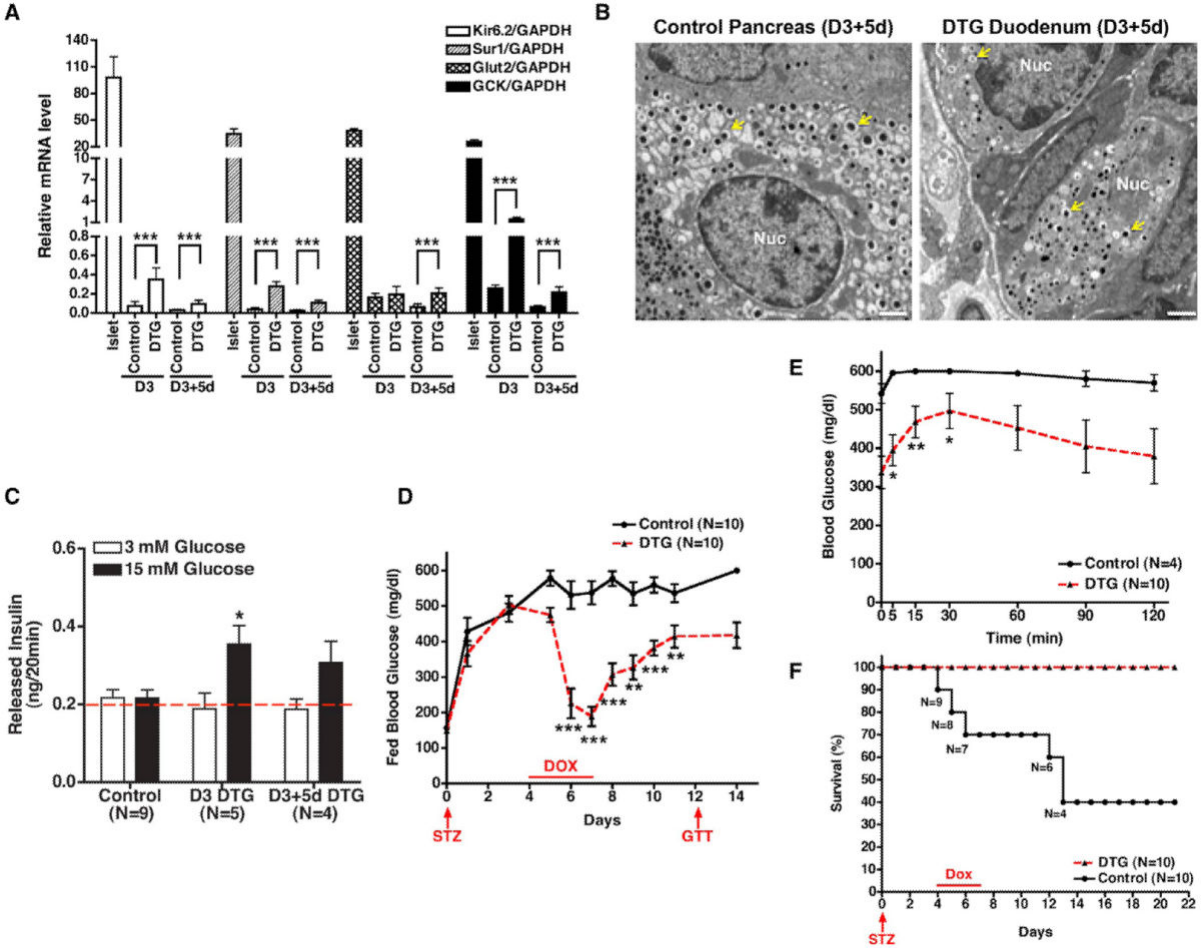
Physiological Features of Intestinal Insulin^+^ Cells (A) Quantitative PCR analysis of the b cell transcripts *Kir6.2*, *Sur1*, *Glut2*, and *glucokinase* (GCK) in normal islets and crypts from control or DTG mice. Crypt cells were isolated from 20 cm segments of mouse small intestines as described in Experimental Procedures. ***p < 0.001, Student's t test. (B) Electron micrographs of a pancreatic b cell from a control mouse and an intestinal crypt cell from D3+5d DTG mouse. b-granules (yellow arrows) can be seen in both. Nuc, nucleus. (C) Glucose-stimulated insulin secretion from intestinal crypt cells. Crypts were isolated from control or DTG intestines as indicated. Insulin was measured by ELISA in the presence of 3 mM or 15 mM glucose for 20 min. The red dashed line (0.2 ng) reflects the background level of the assay (buffer alone). p = 0.0286 by one-way ANOVA between three groups (control, DTG 3 mM glucose, and DTG 15 mM glucose). *p < 0.05. (D) Blood glucose levels of control and DTG animals treated with streptozotocin (STZ) and Dox. Four days after STZ injection, mice were given Dox for 3 days. On day 12 (5 days after Dox withdrawal), mice were subjected to glucose-tolerance testing (GTT). *p < 0.05, **p < 0.01, ***p < 0.001, Student's t test. (E) Blood-glucose levels of control and DTG mice treated with STZ and Dox and challenged with an intraperitoneal injection of glucose (GTT) at day 12 (see D). (F) Kaplan-Meier analysis of control and DTG mice treated with STZ. p < 0.001 between groups. Data are presented as mean ± SD in (A) and mean ± SEM in (C)–(F). The scale bars represent 1 μm. See also Figure S4.

We next sought to determine the extent to which the insulin^+^ cells in DTG intestines resembled normal islet β cells. We began by quantifying the expression of several β-cell-enhanced genes in crypt preparations taken from DTG or control animals. Transcripts for several genes—including the *kir6.2* and *Sur1* subunits of the pancreatic β cell K_ATP_ channel, glucose transporter *Glut2* glucokinase (*GCK*), and *Ins2* itself—were induced in DTG crypts in both the acute and deinduced state, although transcript abundance was dramatically lower compared to purified islets (Figures 4A and S4B). Moreover, quantification of transcript levels for *Nkx2.2* and *Nkx6.1*—two transcription factors important for β cell identity and function—revealed that *Nkx2.2*, but not *Nkx6.1*, was increased (Figure S4A). Earlier report suggested that depletion of FoxO1 induces insulin expression in gut cells (Talchai et al., 2012). We therefore measured the *FoxO1* transcript levels in GFP^+^ crypt cells but observed no difference between DTG and control cells (Figure S4C). Interestingly, we found two CpG sites (−182 and −171) within the *Ins2* promoter that were less methylated in sorted GFP^+^ DTG crypt cells compared to control cells (Figure S4D). Notably, the relative decrease in *Ins2* methylation (~ 30%; Figure S4D) correlated well with the percentage of GFP^+^ cells that were copositive for insulin in these samples (also ~30%), suggesting that promoter demethylation is a rate-limiting step for insulin expression. In islet β cells, insulin is processed and stored in secretory granules that appear ultra-structurally as an electron-dense core surrounded by a clear halo. These dense-core secretory granules are critical for insulin secretion in β cells in response to glucose stimulation. Using electron microscopy, we found intestinal crypt cells from D3+5d DTG mice that contained this hallmark of β cell ultrastructure, whereas such granules could not be found in control crypt cells (Figure 4B).

As the neoislet cells were “mono” hormonal and equipped with glucose-sensing and insulin-releasing machinery (Figures 3, 4A, and 4B), we sought to determine whether these insulin^+^ intestinal cells exhibit glucose-responsiveness. Normal mouse islets do not secrete insulin when exposed to 3 mM glucose, whereas half-maximal stimulation is achieved at ~11 mM glucose (Hedeskov, 1980). Hence, we exposed crypts from control, D3, or D3+5d animals to 3 mM or 15 mM glucose and measured insulin release. Whereas control crypts had a background signal that did not differ between low and high glucose, crypts from DTG mice (D3 and D3+5d) exhibited a significant increase in insulin secretion in 15 mM glucose compared to 3 mM glucose (Figure 4C). These findings suggest that the crypt cells from DTG mice secrete insulin in a glucose-sensitive manner.

Next, we determined whether the intestinally derived insulin^+^ cells could function in place of islet β cells to maintain glucose homeostasis. To this end, we rendered mice diabetic with the β cell toxin streptozotocin (STZ) and tested the effects of PMN induction on survival and glucose homeostasis. Four days after injection of a single dose of STZ, all animals exhibited a marked elevation in nonfasting BG (~450–500 mg/dl as compared to ~150 mg/dl before treatment). Dox was then administered for 3 days to induce the PMN factors. In Dox-treated DTG mice, BG levels returned to normal (mean 134 mg/dl; Figure 4D), a recovery that was associated with normalized serum insulin levels (Figure S4E). In contrast, BG of Dox-treated control mice remained high (>500 mg/dl) for the duration of the experiment (Figure 4D).

Although BG levels in DTG mice began to rise following deinduction (Figure 4D), STZ-treated DTG animals on day 12 exhibited a markedly better response to glucose challenge than controls (Figures 4D and 4E). This improvement was associated with better survival for animals with STZ-induced diabetes, with 100% of DTG animals surviving 2 weeks after deinduction as compared to only 40% of control animals (Figure 4F). The improved glucose homeostasis in diabetic DTG mice at this time point was not due to pancreatic β cell regeneration, as islet β cells were barely detectable in either control or DTG animals 2 weeks after STZ treatment whereas insulin^+^ neoislets were still present at this stage (data not shown). These results indicate that functional insulin-producing glucose-responsive cells persist in the intestine after exogenous PMN expression has ceased.

### Intestinal Insulin^+^ Cells Originate from Crypt Cells and Ngn3^+^ Cells

**Figure 5:**
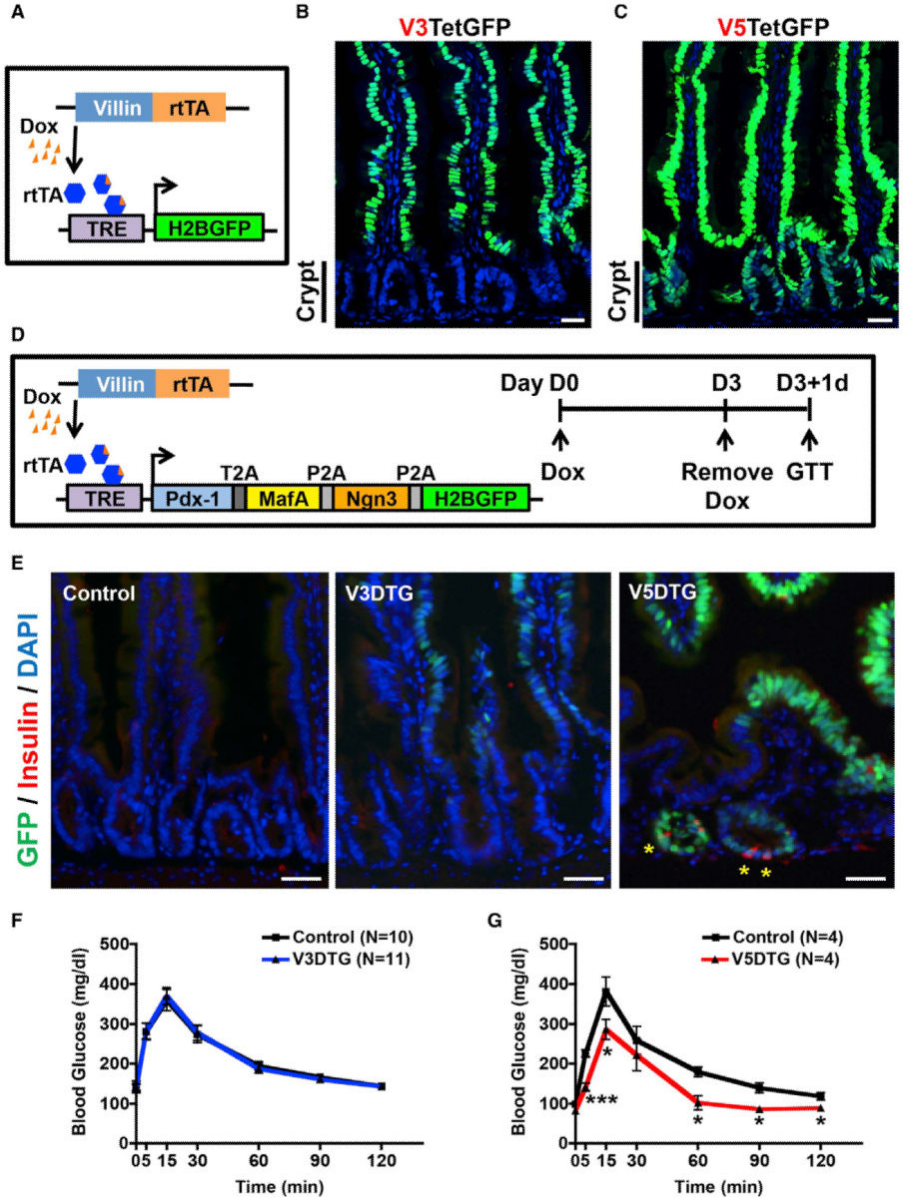
Intestinal Insulin^+^ Cells Are Derived from Crypts (A) Schematic of Villin-rtTA transgene experiments. Different founder of *Villin-rtTA* mouse lines were bred with Tet-GFP mice to obtain V3TetGFP or V5TetGFP mice. (B) The V3-rtTA transgene drives GFP expression in villi, but not crypts, after Dox treatment. (C) The V5-rtTA transgene drives GFP expression throughout the crypt-villus axis after Dox treatment. (D) Schematic of experimental design. V3 and V5-rtTA mouse lines were bred to *R26Tetß* mice to generate V3DTG and V5DTG mice, respectively. V3DTG, V5DTG, and littermate control mice were given Dox for 3 days and then fasted for 17 hr prior to intraperitoneal (i.p.)-GTT assay. Intestinal tissues were collected after i.p.-GTT experiments.

To determine whether neoislets were derived from intestinal epithelial cells, we generated a panel of transgenic mouse lines that express rtTA*M2 under control of the *Villin* promoter (Villin-rtTA*M2), a well-characterized promoter element that directs broad expression within the intestinal epithelium (el Marjou et al., 2004; Madison et al., 2002; Pinto et al., 1999). Using a DNA construct in which a 13 kb regulatory region of mouse *Villin* was placed upstream of rtTA*M2 coding sequences, we created five transgenic Villin-rtTA*M2 founder lines. When crossed to Tet-H2BGFP reporter mice (Tumbar et al., 2004), two of these lines—V3 and V5—yielded distinct epithelial patterns of GFP expression in the presence of Dox (Figure 5A). Specifically, GFP fluorescence was observed throughout the crypt-villus axis in V5TetGFP (Villin-rtTA*M2^V5^; *Tet-H2BGFP*) mice but was limited to the villi of V3TetGFP (Villin-rtTA*M2^V3^; *Tet-H2BGFP*) mice (Figures 5B and 5C). We reasoned that these unique patterns of transgene activity might permit us to determine whether the intestinal insulin^+^ cells are derived from the crypt compartment.

Villin-rtTA*M2 (V3 or V5) mice were bred to *R26Tetß* mice, and the resulting V3DTG and V5DTG mice were treated with Dox for 3 days. Immunofluorescence for insulin revealed that V5DTG, but not V3DTG or control mice, harbored intestinal insulin^+^ cells (Figure 5E). Consistent with these results, V5DTG mice had an improved response to glucose challenge whereas V3DTG mice responded normally (Figures 5F and 5G). These results suggest that the ectopic insulin^+^ cells are derived from epithelial precursors that reside in the intestinal crypts.

We hypothesized that some of these insulin^+^ crypt cells might originate from enteroendocrine progenitor cells that express the basic helix-loop-helix protein Ngn3 (Jenny et al., 2002; Johansson et al., 2007; Schonhoff et al., 2004; Wang et al., 2009). To test this hypothesis, we employed a lineage-tracing strategy to mark endocrine progenitor cells and determined whether they give rise to intestinal insulin^+^ cells. We crossed *Ngn3CreER* mice, which carry a tamoxifen (TM)-inducible Cre recombinase gene under the control of the Ngn3 promoter (Gu et al., 2002) to *R26Cherry* mice, which carry a lox-STOP-lox cassette upstream of an H2B-mCherry reporter gene, resulting in bigenic NC mice (Figure S5A). As expected from previous reports (Jenny et al., 2002; Schonhoff et al., 2004), Cherry-labeled cells were observed in crypts and scattered villi following two injections of TM (Figure S5B).

**Figure 6:**
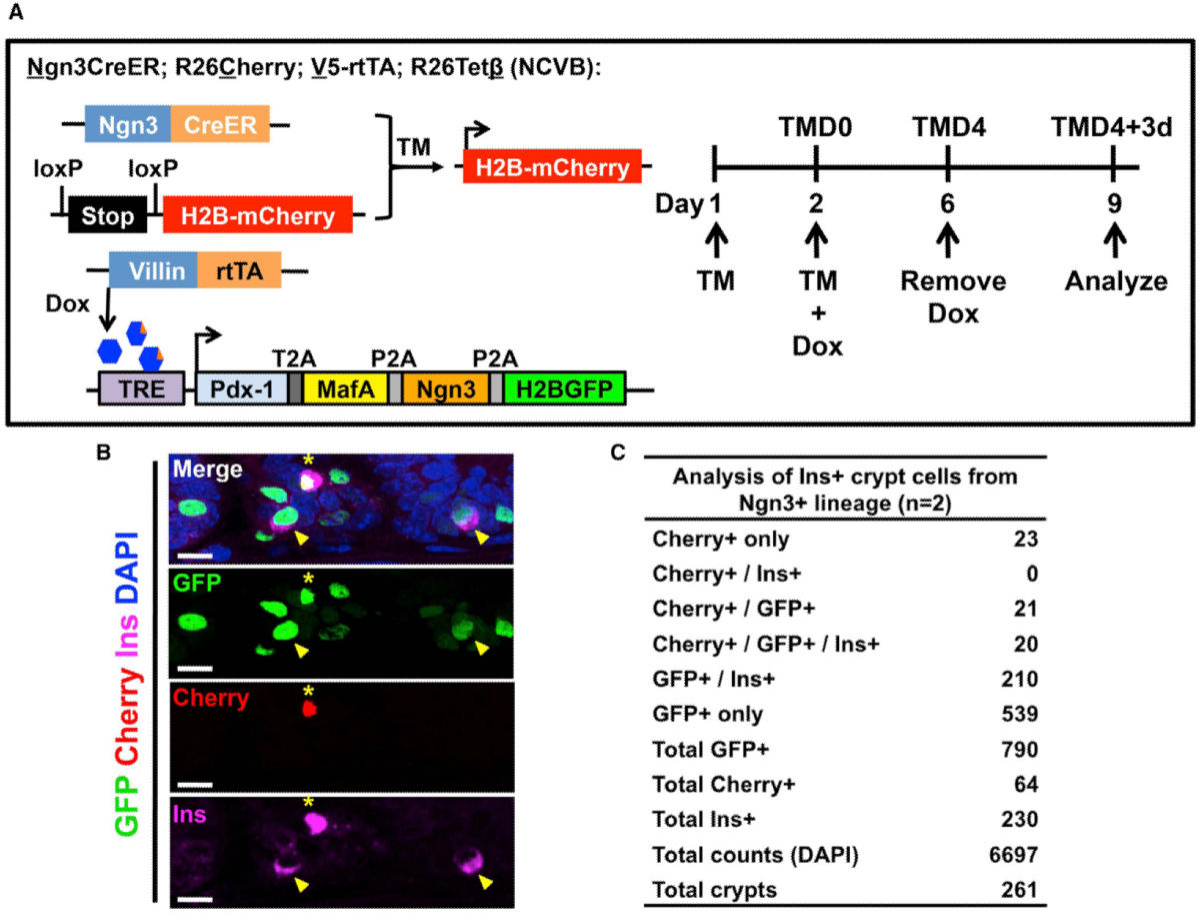
Intestinal Insulin^+^ Cells Can Arise from Ngn3^+^ Cells (A) Schematic of Ngn3-lineage-tracing experiment in insulin^+^ crypt cells. V5DTG mice were bred to *Ngn3CreER* and *R26Cherry* reporter mice to generate NCVB mice. Four- to five-week-old NCVB mice were injected twice with 8 mg tamoxifen (TM) to label the Ngn3^+^ cells. Mice were then given Dox-containing water for 4 days to induce intestinal PMN expression followed by 3 days of untreated water prior to analysis. (B) Representative IF images of NCVB intestines stained for insulin and DAPI. The asterisk indicates a GFP^+^/insulin^+^ cell that carries the mCherry Ngn3 lineage label, whereas arrowheads indicate GFP^+^/insulin^+^ cells that are negative for mCherry. The scale bars represent 10 μm. (C) Quantification of Ngn3-lineage-traced insulin^+^ crypt cells. See also Figure S5.

We then introduced the NC alleles into the V5DTG background to generate *Ngn3CreER*, *R26Cherry*, *V5-rtTA*, and *R26Tet*β (NCVB) mice. NCVB mice were given two injections of TM to label Ngn3^+^ endocrine progenitors, treated with Dox for 4 days to induce intestinal expression of the PMN factors, and then examined 3 days later for expression of GFP, mCherry, and insulin (Figure 6). A total of 6,697 crypt cells were counted, of which 790 stained for GFP and 64 stained for mCherry (Figure 6C). Consistent with our prior measurements, 29.1% of the GFP^+^ cells costained for insulin. Notably, 8.7% of these GFP^+^/Ins^+^ cells were also positive for mCherry, indicating they had originated from Ngn3^+^ cells. We then examined the mCherry^+^ cells and found that two-thirds of them (41/64) were GFP^+^, indicating efficient expression of the Tetβ transgene within this pool of cells. Significantly, 20 out of these 41 GFP^+^/mCherry^+^ cells also expressed insulin (Figure 6C), indicating that PMN expression in an Ngn3-marked cell is associated with a nearly 50% chance of the cell becoming insulin^+^. Taken together, these data suggest that at least some of the intestinal insulin^+^ cells observed in Tetb mice are progeny of Ngn3^+^ endocrine progenitor cells. However, because *Ngn3CreER* does not result in labeling of all endocrine progenitors, we cannot conclude whether all or only a fraction of the intestinal insulin^+^ cells came from this source.

### Generating Insulin^+^ Cells from Human Intestinal Organoids

We next sought to determine whether human intestinal cells are capable of acquiring β-like characteristics under the influence of PMN. To this end, we used human ESC (hESC)-derived intestinal organoids to generate three-dimensional structures containing Lgr5^+^/Ascl2^+^ crypt-like domains in vitro (McCracken et al., 2011). Importantly, these structures recapitulate many of the features of normal human intestinal tissue, including an intact crypt-villus axis and differentiation of all intestinal lineages (Spence et al., 2011).

**Figure 7:**
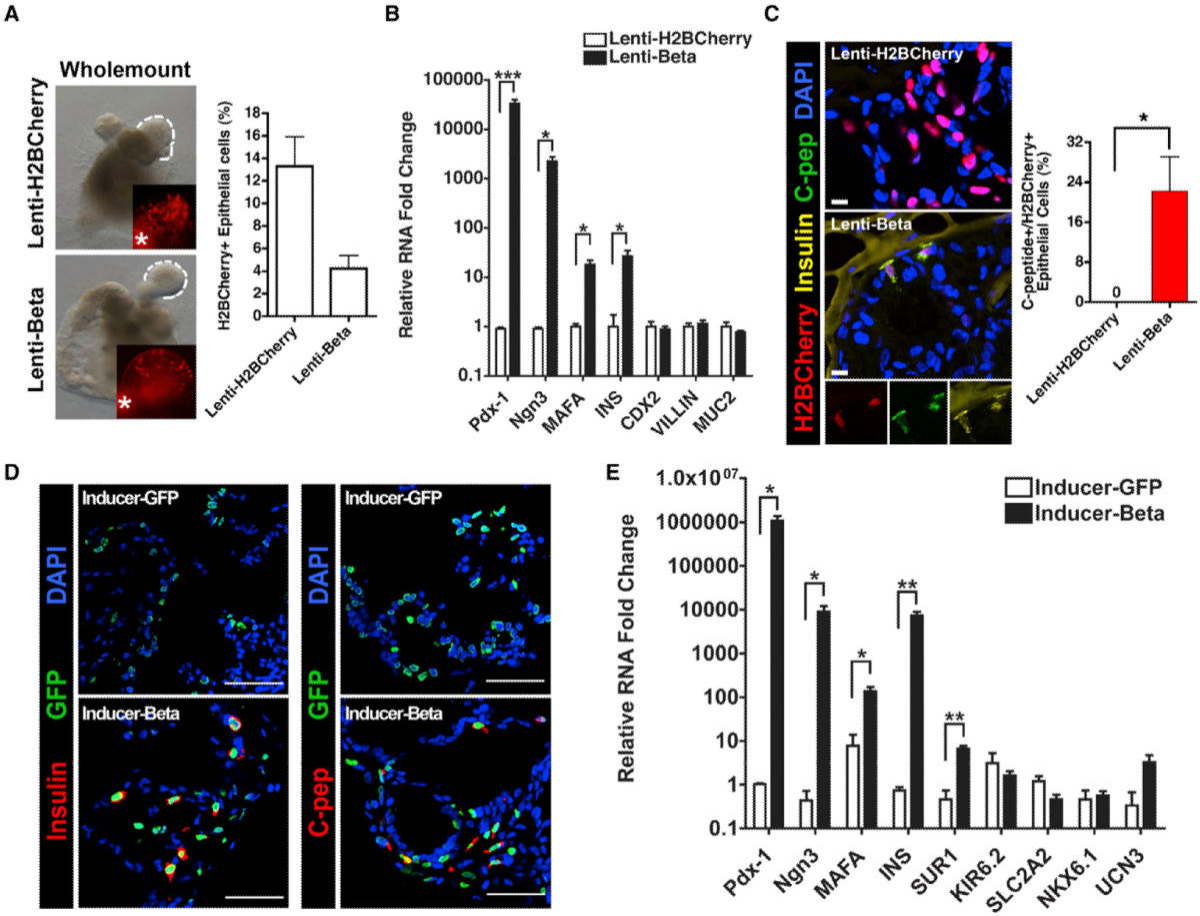
Insulin Production in Human Crypt Cells following PMN Expression (A) Whole-mount bright field and immunofluorescence images from human intestinal crypt organoids (HIOs) infected with lenti-H2BCherry or lenti-beta virus. Infection rates were calculated by counting H2B-Cherry^+^ cells as a percentage of epithelial cells in the organoids. (B) Quantitative PCR analysis of HIOs after infection with lenti-H2BCherry or lenti-beta virus for the indicated transcripts compared to GAPDH control. Experiments were performed using biological triplicates (each biological group contained at least five organoids). *p < 0.05, Student's t test. (C) Immunofluorescence for H2BCherry, insulin, and C-peptide in lenti-H2BCherry- or lenti-beta-infected organoids. Insulin^+^/C-peptide^+^ cells were detected exclusively in lenti-beta-infected human crypt organoids and quantified (right). *p < 0.05, Student's t test. (D) Immunofluorescence for insulin and C-peptide in inducible HIOs. Inducible HIOs were generated from Inducer-GFP or Inducer-beta-virus-infected hESCs. Insulin and C-peptide signals were detected and colocalized with GFP^+^ cells in Inducer-beta HIOs. (E) Quantitative PCR analysis of inducible HIOs for the indicated transcripts compared to GAPDH control. Experiments were performed using biological triplicates (each biological group containing at least five organoids). *p < 0.05, **p < 0.01, Student's t test. Data are presented as mean ± SEM. The scale bar represents 10 μm (C) and 50 μm (D). See also Figure S6.

Infection of human intestinal organoids (HIOs) with either lenti-beta or lenti-H2BCherry viruses resulted in the appearance of H2BCherry^+^ cells with an efficiency ranging from 4% to 13% (Figure 7A). Analysis of RNA from infected HIOs revealed a dramatic increase of transgene (*Pdx-1*, *Ngn3*, and *MafA*) as well as human *INS* transcripts in lenti-beta-infected HIOs as compared to lenti-H2BCherry-infected HIOs, with no change in transcript levels for *CDX2, VILLIN*, and *MUC2* (Figure 5B). Confocal IF for H2BCherry, insulin, and C-peptide confirmed that lenti-beta-infected cells, but not Lenti-H2BCherry-infected cells, expressed insulin as well as C-peptide (Figure 7C). Specifically, approximately 22% of the lenti-beta-infected cells stained for human C-peptide, whereas none of the lenti-H2BCherry-infected cells did (Figure 5C).

To achieve uniform temporal and reversible control of PMN genes expression in hESC-derived HIOs, we generated the pInducer-GFP and pInducer-beta viruses from an inducible lenti-vector system (Meerbrey et al., 2011; Figure S6A). Human ESCs were infected with either pInducer-beta or pInducer-GFP virus, and neomycin selection was applied to establish stable cell lines, which in turn were used to generate Inducer-beta or Inducer-GFP HIOs. After 1 month of organoid formation, the transgenes were induced by 10 days treatment with Dox followed by IF and RNA analysis. Following this treatment, GFP^+^ clusters were readily seen in Inducer-beta HIOs, similar in appearance to the neoislets we observed in DTG mice in vivo (Figure S6B). IF analysis demonstrated robust expression of Pdx-1, Ngn3, and ChroA in the GFP^+^ cells (Figures S6C and S6D); moreover, insulin and C-peptide staining was present in a large fraction of the cells (Figures 7D, S6C, and S6D). RNA analysis revealed significant induction of human *INS* and *SUR1* transcripts, but no increase in *KIR6.2* or *NKX6.1*. These results suggest that, within human organoids—an in vitro environment that recapitulates features of the normal human intestine—PMN expression enables a β-like conversion similar to the one observed in the DTG mouse model.

## DISCUSSION

### Generating Insulin^+^ Cells in the Intestine

We have conducted an in vivo screen for adult cell types that are competent to become insulin-producing cells. We found that ectopic expression of Pdx1, MafA, and Ngn3 in the intestinal crypts, but not villi, results in their conversion into cells with β-like features. These include the production and processing of insulin, formation of cytoplasmic β-granules, and expression of components of the glucose-sensing machinery. Importantly, these insulin^+^ crypt cells can sense glucose levels and release insulin in response to high glucose, a property that can ameliorate the hyperglycemia caused by STZ-induced β cell ablation in vivo. Finally, these changes in cell phenotype are reproduced in human intestinal organoids, suggesting that the human intestine may also be competent to give rise to functional insulin-producing cells.

Adenoviral delivery of one or more PMN factors in either hepatocytes (Ferber et al., 2000; Kaneto et al., 2005; Yechoor et al., 2009) or pancreatic acinar cells (Zhou et al., 2008) can promote the formation of insulin^+^ cells in vivo. DTG mice failed to express the PMN factors in the liver, and thus the *R26Tet*ß system could not be used to assess the competence of hepatic cells to become insulin^+^ cells with these three genes. This lack of hepatic expression was not due to a failure of the Tet-On system, because GFP was readily detectable in the liver of Dox-treated R26-rtTA*M2; Tet-GFP mouse (Figure S1D). This suggests that other factors or negative regulators in the liver are responsible for silencing of the PMN polycistronic mRNA.

By contrast, we observed robust expression of the PMN factors in the exocrine pancreas. Despite this, we were unable to detect insulin or ChroA staining in the acinar cells of DTG mice. There are several potential explanations for the discrepancy between our results and previous studies reporting “acinar reprogramming” (Akinci et al., 2012, 2013; Zhou et al., 2008). First, the adenoviral delivery method employed by these studies may have established an inflammatory microenvironment that was more conducive to cellular transdifferentiation, as has been suggested (Lee et al., 2012; Wang et al., 2007). Alternatively, the in vivo conversion of acinar cells to β-like cells (Zhou et al., 2008) may have required a specific stoichiometry of PMN expression that was not achieved in the *Tet*ß system. Such a dependence on stoichiometry has been noted in studies using polycistronic vectors to create iPSCs (Carey et al., 2011). Despite this difference in cellular competence, the morphogenetic and transcriptional changes that we observed in the intestine occur with a rapidity that echoes the observations of Zhou et al. After only 3 days of PMN expression, GFP^+^/insulin^+^ cells were abundant in intestinal crypts and villi. Five days later (after deinduction), these cells had coalesced as neoislets. Moreover, whereas a subset of GFP^+^ cells coexpressed glucagon during the induction period, insulin was the only hormone detected in neoislets.

### Origins of Intestinal Insulin^+^ Cells

Which cells within the intestinal epithelium give rise to neoislets? We have shown that neoislets emerge principally from the duodenum and proximal jejunum, sections of the midgut that are in close apposition to prepancreatic endoderm during development. Hence, we speculate that this portion of the intestine retains competence to give rise to β-like cells by virtue of its embryonic heritage.

Using an allelic series of *Villin-rtTA* driver strains, we demonstrated that neoislets are derived from the intestinal crypts rather than villi. Furthermore, using a combination of rtTA-mediated misexpression and Cre-mediated lineage tracing, we found that at least some of the neoislet cells are derived from endocrine progenitor cells. Others (Cheung et al., 2000) have previously shown that mouse intestinal K cells are capable of correctly processing human insulin. Although our lineage-tracing studies cannot exclude the possibility that some of the insulin^+^ cells are derived from differentiated cells, such as K cells, our results are more reminiscent of the findings of Talchai and colleagues (Talchai et al., 2012), who reported that deletion of *FoxO1* in gut Ngn3^+^ cells resulted in the emergence of intestinal insulin^+^ cells. Importantly, there was no difference in FoxO1 expression when we compared DTG and control crypt cells (Figure S4C), suggesting that FoxO1 lies upstream of the PMN factors or that the two genetic manipulations promote the formation of insulin^+^ cells by independent mechanisms. Given that Ngn3 progenitors comprise only ~1% to 2% of cells within the crypts, and neoislets emerge rapidly and robustly in the absence of significant cell division (Figure S3), it remains possible or even likely that other cell types in addition to Ngn3^+^ progenitors contribute to neoislet formation.

### Functionality of Intestinal Insulin^+^ Cells

Although the neoislets in DTG intestines are unlikely to contain fully functional β cells, several lines of evidence suggest that these cells have “β-like” properties that could prove useful. First, neoislet cells are monohormonal and have morphological and molecular hallmarks of β cells, including the ability to process preproinsulin into its mature form (with release of C-peptide), up-regulation of the genes encoding the β cell K_ATP_ channel subunits *Kir6.2* and *Sur1*, and the presence of distinctive β-granules. Furthermore, D3+5d animals have improved glucose tolerance with no decrease in fasting blood glucose, suggesting that the neoislets function in a regulated fashion. Neoislets conferred a marked survival benefit in diabetic animals, and even several days after transient expression of the PMN factors had ceased, STZ-treated diabetic DTG animals retained a better response to glucose challenge than nontransgenic animals. Finally, crypts isolated from D3 or D3+5d Dox-treated animals secreted insulin in response to high, but not low, concentrations of glucose.

Nevertheless, neoislet cells gradually disappear from DTG intestines after deinduction (~2 to 3 weeks). One possible explanation is that ectopic PMN factors are not sufficient to establish a stable β-like state, a status that might require additional cooperating transcription factors. One attractive candidate for such a “missing” factor is Nkx6.1, as this β-cell-enriched transcriptional regulator was not induced by PMN in vitro or in vivo. Nkx6.1 is essential for the establishment of β cell identity during development (Schaffer et al., 2013) and appears to be necessary for β cell maturation (Rezania et al., 2013; Taylor et al., 2013), a role that is compatible with the immature phenotype we observe in DTG neoislets. These issues of stability and functionality will need to be addressed before neoislets can be considered for diabetes therapy.

Full-fledged cellular reprogramming, as occurs in the conversion of differentiated fibroblasts to a pluripotent state, requires the formation of stable feedforward transcriptional networks, a process that involves marked chromatin remodeling (Apostolou and Hochedlinger, 2013; Buganim et al., 2013). Our organoid experiments suggest that the expression of exogenous PMN factors (mouse origin) does not result in the induction of their endogenous (human) counterparts (PDX-1 and NGN3). Because the intestinal insulin^+^ cells have not achieved such a transcriptional rewiring, we do not believe they represent fully “reprogrammed” β cells. Nevertheless, we propose that the PMN factors do cause a “partial” reprogramming, as our finding of decreased *Ins2* promoter methylation represents evidence for epigenetic rewiring of the cells. Our studies thus create a framework for the identification of additional factors that can promote “complete” reprogramming of fully functional β cells from the intestinal epithelium.

Over the past decade, there has been major interest in identifying cell populations that can be coaxed into becoming pancreatic β cells. Our results provide strong evidence that the intestine, one of the largest tissues of the body, is an appealing candidate. Moreover, our findings with human intestinal organoids suggest that the human intestinal cells might preserve the ability to form β-cell-like neoislets. Given the almost unlimited potential for expansion of human intestinal tissue in culture (Sato et al., 2011), our observation that ectopic expression of PMN causes human intestinal organoids to adopt β-like features suggests that it may be possible to exploit and extend these findings in humans, potentially for therapy.

## EXPERIMENTAL PROCEDURES

### Mouse Strains

The following mouse strains were utilized in this study: *R26Tetß*, *Villin-rtTA* (*V3* and *V5*), R26rtTA*M2 (Hochedlinger et al., 2005), *Tet-H2BGFP* reporter mice (Tumbar et al., 2004), and *Ngn3CreER*™ (Gu et al., 2002). R26rtTA*M2 and TetGFP reporter mice were obtained from Jackson Lab. *R26Cherry* mice were made by targeting a CAGS-lox-PGK-neo-p(A)-lox-H2BCherry p(A) cassette into ROSA26 locus in V6.5 ESCs (kindly provided by Dr. Doug Melton). Methods for making the *R26Tetß* and *Villin-rtTA* mouse strains are detailed in the Supplemental Experimental Procedures. Both male and female mice at 4–6 weeks of age were used for all experiments; post hoc analysis revealed no gender effect on the experimental results. To induce PMN gene expression, DTG and V3DTG or V5DTG mice were fed 0.2 mg/ml or 2 mg/ml doxycycline (Dox; Sigma), respectively, in the drinking water supplemented with 20 mg/ml sucrose. Tamoxifen (Sigma) was dissolved in corn oil at 40 mg/ml. Four- to five-week-old NCVB mice were given 8 mg tamoxifen on consecutive days prior to treatment with 2 mg/ml Dox. All animal experiments were performed in accordance with the National Institutes of Health policies on the use of laboratory animals and approved by the Institutional Animal Care and Use Committee of the University of Pennsylvania.

### Antibodies and Immunohistochemistry

Tissues were fixed in 4% paraformaldehyde overnight at 4° C. Subsequently, tissues were washed three times in PBS, incubated in 15% sucrose solution for 2~3 hr and then 30% sucrose overnight (12~16 hr) before optimum cutting temperature embedding for cryosection. A detailed description of primary antibodies used in IF staining and western blot analysis is provided in the Supplemental Experimental Procedures (Table S2). Rhodamine-Red-X-conjugated and Alexa Fluor-conjugated donkey secondary antibodies were obtained from the Jackson Immunoresearch Laboratories. Immunofluorescence images were taken with either a Zeiss LSM 710 Confocal microscope or an Olympus IX71 fluorescent microscope.

### Crypt Cell Isolation

Crypt cells were isolated as described previously (Sato et al., 2009), with modifications. A detailed description is provided in the Supplemental Experimental Procedures. For quantitative PCR (qPCR) experiments, crypt cells were pooled from intestines of two mice. For insulin secretion studies, each crypt sample was isolated from a single mouse.

### Insulin Secretion Assays

Freshly isolated small-intestinal crypts from individual mice were divided equally between two tubes and washed three times with warm Krebs-Ringer bicarbonate buffer (KRBB; 115 mmol/l NaCl; 24 mmol/l NaHCO_3_; 5 mmol/l KCl; 1 mmol/l MgCl_2_; 2.5 mmol/l CaCl_2_; 10 mM HEPES [pH 7.4]) without glucose and supplemented with 0.25% BSA. The cells were then incubated with KRBB containing either 3 mM or 15 mM glucose for 20 min at 37° C. Supernatant was collected, and insulin was measured by homogenous time-resolved fluorescence insulin assay using mouse insulin as a standard (Cisbio). Three independent experiments were performed to analyze insulin secretion ability in crypt cells.

### Transcriptional Analysis

Total RNAs from mouse tissues and crypt cells were purified using TRIzol reagent (Invitrogen) followed by DNaseI (Promega) digestion. Equal quantities of DNaseI-treated RNA were reverse transcribed using an iScript cDNA synthesis kit (Bio-Rad). The cDNAs were subjected to conventional PCR method or real-time qPCR. The qPCR reactions were amplified and analyzed in triplicate using CFX384 Real-Time PCR detection system (Bio-Rad). All experiments were repeated twice. Primers and annealing temperatures (Ta) used for amplification were listed in Supplemental Experimental Procedures (Table S1). A detailed description of quantitative RT-PCR (qRT-PCR) on human intestinal organoids was provided in the Supplemental Experimental Procedures.

### Physiological Studies

A single injection of STZ (200 mg per g body weight) was administrated intraperitoneally to induce diabetes in 4- to 5-week-old mice. Mice that were not hyperglycemic (fed BG > 400 mg/dl) 3 days after STZ administration were excluded from further study. Diabetic animals were fasted overnight (12 hr) and then given Dox water (0.2 mg/ml doxycycline and 2% sucrose) on day 4 for 3 days. Fasting BG was measured with a One Touch Ultra Glucometer (LifeScan) after 16-18 hr fast. Fed BG levels were measured in the late morning (10 am-noon) after a 1 hr fast. Glucose tolerance testing was performed after an overnight fast (16-18 hr), followed by intraperitoneal injection of glucose (3 g per kg body weight). At the indicated time points, BG was measured and blood was collected in microvette CB 300 (SARSTEDT) tubes for detection of serum insulin levels using an Ultrasensitive Mouse Insulin ELISA kit (Crystal Chem).

### Electron Microscopy

Dissected duodenum and pancreas were fixed immediately at 4° C overnight in 2.5% glutaraldehyde and 1% paraformaldehyde. Samples were rinsed with 0.1 M sodium cacodylate, postfixed with 1% osmium tetoxide, dehydrated with ethanol, and embedded in Epon resin. Semithin sections (1 μm) stained with toloudine blue were used to identify neoislet structures in the DTG duodenum by comparing with wild-type duodenum. Regular ultra-thin sections were prepared for electron microscopic examination of granules. Digital images were acquired with a JEOL-1010 transmission electron microscope.

### Human Intestinal Organoid Culture

Human ESC-derived intestinal organoids (HIOs) were generated and maintained as previously described (McCracken et al., 2011; Spence et al., 2011), with some modifications. A detailed description of HIOs and inducible HIO culture is provided in the Supplemental Experimental Procedures. Briefly, HIOs were infected with virus twice and then harvested 15 days after infection for IF and qRT-PCR analysis. Inducible HIOs were treated with 2 μg/ml of doxycycline for 10 days then collected for IF and RNA analysis. Each HIOs experiment included at least three independent biological replicates, and each biological replicate consisted of a pool of 3–5 organoids. All work involving human pluripotent (embryonic) stem cells was reviewed and approved by the University of Michigan Human Pluripotent Stem Cell Research Oversight committee.

### Statistical Analysis

p values were calculated by Student's t test. One-way ANOVA analysis was used in the insulin secretion assay to compare multiple groups. Error bars reflect SEM or SD as described in each individual figure legend.

## Supplementary Material

01
