# Supplementary material for: De Novo Formation of Insulin-Producing “Neo-β Cell Islets” from Intestinal Crypts
Source: Cell Rep. Author manuscript; Available in PMC 2014 Nov 26. (PMC4245054; doi:10.1016/j.celrep.2014.02.013)
Supplement: 01 [file NIHMS644280-supplement-01.pdf]

Supplemental Information

De novo formation of insulin-producing "neo-ß-cell islets" from intestinal crypts

Yi-Ju Chen[1,3], Stacy R. Finkbeiner[9], Daniel Weinblatt[1,3], Matthew J. Emmett[1,2], Feven Tameire[4], Maryam Yousefi[2], Chenghua Yang[1,3], Rene Maehr[6], Qiao Zhou[7], Ruth Shemer[8], Yuval Dor[8], Changhong Li[5], Jason R. Spence[9,10,11], and Ben Z. Stanger[1,2,3]*

Supplemental Experimental Procedures

Vectors and viral preparation.

To generate pLenti-Beta – a polycistronic cassette encoding *Pdx1*, *MafA*, *Ngn3* and *H2B-Cherry* – was cloned into the FUW lentivirus backbone using XbaI and AscI. The polycistronic cassette was generated using unique restriction sites, resulting in the placement of individual factors between 2A peptide sequences as follows: T2A, 5'-GAGGGCAGAGGAAGTCTTCTAACATGCGGTGACGTGGAGGAGAATCCCGGC CCT-3' and P2A, 5'-GCCACGAACTTCTCTCTGTTAAAGCAAGCAGGAGACGTGG AAGAAAACCCCGGTCCC-3'. H2B-mCherry cDNA was amplified by PCR and cloned into XbaI and AscI sites on the FUW lentivirus backbone to generate the pLenti-H2BmCherry plasmid. The inducible Lenti-vectors (pInducer-GFP and pInducer-Beta) were made using the pENTR Directional TOPO Cloning method with pInducer20 destination vector. The pInducer20 vector is an inducible Lenti-vector system, which carries both rtTA3 and Neomycin-resistance genes under the UBC promoter as well as a cDNA of interest under the control of a tetracycline-responsive promoter (Meerbrey et al., 2011). First, H2BGFP cDNA or PMN-H2BGFP polycistronic cassettes were PCR amplified and subcloned into pENTR/D-TOPO vector (Invitrogen), respectively. The pInducer-GFP and pInducer-Beta vectors were obtained after performing the LR recombination and confirmed by sequencing. All lentiviruses were generated by co-transfecting the viral vector with three packaging plasmids (pVSVG, pRSV, and pMDL) into 293T cells using Lipofectamine 2000 (Invitrogen). Viral supernatants were collected

48h or 72h after transfection, filtered through a 0.22-uM filter and stored at -80 °C. An immortalized human hepatocyte cell line (HHLS) was transduced with Lenti-Beta virus in DMEM media containing 10% FBS and 8ug/ml polybrene to confirm transgene expression. H2BCherry fluorescence and immunoreactivity for Pdx-1, MafA, and Ngn3 were detected 72h post-infection.

Generating the R26Tetß mouse strain.

The polycistronic cassette containing Pdx1-T2A-MafA-P2A-Ngn3-P2A was amplified by PCR from pLenti-Beta plasmid and digested with BglII and HindIII. H2B-GFP was amplified by PCR followed by HindIII and NotI digestion. The TRE-tight promoter was obtained by digesting the pTRE-tight (Clontech) plasmid with XhoI and BamHI. All three PCR products were cloned together into an XhoI/NotI-digested pBigT vector to create the PGKneo-Tet-Beta-H2BGFP construct, which was sequence verified. To make the *R26Tetß* targeting construct, the PGKneo-Tet-Beta-H2BGFP cassette was inserted into PacI and AscI sites of the pRosa26PA targeting vector. To generate *R26Tetß* ES cells, the pRosa26PA-PGKneo-Tet-Beta-H2BGFP targeting plasmid was linearized with SwaI and electroporated into V6.5 ES cells, and stable integrants were selected with G418 (250μg/ml). Homologous recombination was confirmed by Southern blotting using a *Rosa26* external probe. Six out of 15 analyzed clones (~40%) were found to have homologous transgene integration as determined by southern blot analysis (data not shown). To confirm Dox-dependent regulation of transgenes and GFP expression in *R26Tetß* ES cells, the cells were transduced with Lenti-rtTA*M2 virus in ES cell media containing 5 ug/ml polybrene. Regular ES cell medium was added 48 hr post-infection in the presence or absence of 1 ug/ml Dox. GFP fluorescence was detected 24h post-Dox treatment. Following karyotyping to confirm euploidy, correctly targeted *R26Tetß* ES cells were injected into C57BL/6 blastocysts and the resulting chimeric mice were screened for germline transmission. *R26Tetß* mice were bred to *R26-rtTA*M2* mice to obtain (*R26-rtTA*M2*; *R26Tetß*) DTG mice. DTG mice were given Dox water (0.2mg/ml doxycycline, 2% sucrose) as indicated in the text followed by further physiology test and molecular analysis. The *R26Tetß* strain was maintained by breeding to C57BL/6 wild type mice.

Generating the Villin-rtTA mouse strain.

The Villin-rtTA-M2 construct contains a 13Kb regulatory region of the mouse villin gene (7.3 kb upstream and 5.7 kb downstream from the transcription start site) in front of rtTA*M2 coding sequence (a gift from Dr. Blair Madison)(Madison et al., 2002). The 13.8 kb villin-rtTA*M2 cassette was cut out of the plasmid backbone by SalI and EcoRV digest, gel purified, and prepared for injection into B6SJL/F1 oocytes. Founders were identified by PCR amplification of tail DNA using rtTA*M2 specific primers (rtTA-M2 S: 5'- CTGGGAGTTGAGCAGCCTAC-3' and rtTA-M2 AS: 5'- AGAGCACAGCGGAATGACTT-3'). Transgenic *Villin-rtTA*M2* animals were bred with *Tet-H2B-GFP* mice (Tumbar et al., 2004). The 4-5 wk old *Villin-rtTA*M2*;*Tet-H2B-GFP* mice were provided drinking water containing 2mg/ml doxycycline (Dox) (Sigma, D9891) and 2% sucrose. After 3 days of Dox treatment, mice were sacrificed and tissues were analyzed for H2B-GFP expression. Transgenic *Villin-rtTA*M2* animals were also bred with *R26Tetß* mice. The *Villin-rtTA*M2*; *R26Tetß* mice and their littermate controls were given Dox water (2mg/ml Dox, 2% Sucrose) for 3 days and then fasted overnight prior to IP-GTT assay.

Crypt cell isolation

Crypt cells were isolated as described previously (Sato et al., 2009) with some modifications. Twenty cm segments of small intestine (measured from the pylorus) were isolated from mice of the appropriate genotype, opened longitudinally, and washed with cold PBS. The tissues were minced into small pieces and incubated in 5 mM EDTA with PBS for 5 min at RT. After removal of EDTA buffer, the tissue fragments were vigorously suspended in cold PBS. The supernatant (containing the villous fraction) was discarded, and the sediment (containing the crypts) was resuspended with PBS. These steps were repeated until the supernatant became clear, indicating a pure crypt preparation. The sediment was then resuspended and incubated in 5mM EDTA buffer on ice for another 45 min. After further vigorous pipetting, the sample was passed through a 100-um cell strainer (BD Bioscience) to remove residue and large cellular aggregates, centrifuged at 800 rpm for 3 min to remove single cells, and then centrifuged at 1200 rpm

for 3 min to collect crypts. For qPCR experiments, crypt cells were pooled from intestines of two mice. For insulin secretion studies, each crypt sample was isolated from a single mouse.

Human intestinal organoid culture.

Human intestinal organoids were generated and maintained as previously described (McCracken et al., 2011; Spence et al., 2011) with some modifications. N2 supplement was omitted from the growth media and insulin-free B27 supplement (Invitrogen, #0050129SA) was used in place of regular B27 (Invitrogen, #17504044). Prior to lentiviral infection, organoids were removed from matrigel by tituration using a cut p200 pipette. Organoids were transferred to a petri dish containing Advanced DMEM/F12 and were cut into halves or quarters using a sterile tungsten needle and scalpel. Cut organoids were then transferred to eppendorf tubes containing 1mL of supernatant from control virus (Lenti-mCherry) or Lenti-Beta virus. Organoids were incubated at room temperature with gentle rocking for 8 hours. Organoids were then transferred to fresh viral supernatant in a 24 well tissue culture dish and incubated overnight at 37°C in a 5% CO2 tissue culture incubator. The next morning, organoids were embedded in fresh matrigel, and given organoid growth media. Growth media was changed every 2-4 days, and organoids were harvested 150d after infection. Each human intestinal organoid experiment included at least 3 independent biological replicates, and each biological replicate consisted of a pool of 3-5 organoids.

To generate the inducible HIOs, hES cells were first infected with pInducer-GFP or pInducer-TetBeta virus followed by Geneticin selection (100 ug/mL, Gibco #10131) to establish stable cell lines. Selection was carried out for 21 days prior to using the inducible stable cell lines to create inducible-HIOs. Inducible-HIOs took one month to become established at which point they were treated with 2μg/ml of doxycycline (Fisher, #BP2653-5) for 10 days with media changes every other day and then collected for IF and RNA analysis. All HIO experiments included at least 3 independent biological replicates, and each biological replicate consisted of a pool of 3-5 organoids.

qRT-PCR on human intestinal organoids.

RNA was extracted using a MagMAX RNA Isolation Kit (Life Technologies, #11109052) and cDNA was generated using a SuperScript VILO kit (Life Technologies, #11754050). qRT-PCR was carried out using Quantitect Sybr Green Master Mix (Qiagen, #204141) using primers obtained from qPrimerDepot (Cui et al., 2007) with an annealing temperature of 60°C on a StepOne^{TM} Real-Time PCR System (Life Technologies).

Image Quantification

For acinar quantification of insulin expression, two DTG animals were given Dox for 4d, and pancreas sections from each animal were immunostained for insulin and DAPI. Cells were counted from 10 randomly selected fields from multiple sections, accounting for a total of 5818 cells. To examine insulin^{+} cells within the intestinal crypts, sections from Dox-treated DTG animals (D3, n=2; D3+5d, n=9) were immunostained for insulin and DAPI. Cells were counted from 10 randomly selected fields from multiple sections. For Ngn3-lineage tracing studies, two NCVB animals were used for quantification. Frozen sections containing the 20cm-long intestine were immunostained for insulin and DAPI, and cells were counted from 45 and 50 randomly selected fields along the length of the intestine.

Table S1. List of primer sequences used for RT-qPCR (related to Experimental Procedures)

Primer	Primer Sequence (5'-3')	Ta (°C)
**Primers for mouse samples:**		
mPdx1_F	5'- ATG AAA TCC ACC AAA GCT CA	58
mPdx1_R	5'- GTA GGC AGT ACG GGT CCT CT	
mMafA_F	5'- ATC ACC ATC ACC ACC ATC AC	58
mMafA_R	5'- TTC TCG CTC TCC AGA ATG TG	
mNgn3_S	5'- CGG ATG ACG CCA AAC TTA CA	60
mNgn3_AS	5'- GTT ACC CGC TTG GGA GAC TG	
H2B-GFP_F	5'- AGA ACG GCA TCA AGG TGA AC	58
H2B-GFP_R	5'- TGC TCA GGT AGT GGT TGT CG	
mIns1_F	5'- CCA TCA GCA AGC AGG TCA TTG	58
mIns1_R	5'- TGT GTA GAA GAA GCC ACG CTC C	
mIns2_F	5'- CAG AAG CGT GGC ATT GTA GA	58
mIns2_R	5'- GCT GGT AGA GGG AGC AGA TG	
mGAPDH_F	5'- AAC TTT GGC ATT GTG GAA GG	58
mGAPDH_R	5'- ACA CAT TGG GGG TAG GAA CA	
mKir6.2_F	5'- GTG GGT GGT AAC GGC ATC TT	60
mKir6.2_R	5'- GGT GGT GGT GCA GGT CAC TA	
mSur1_F	5'- CCA GTG CAG AGA TCC GTG AG	60
mSur1_R	5'- CAG GGG TCC AGG TGA AGA AG	
mGlut2_S	5'- AAA AGG AAG AGG CAT CGA CT	60
mGlut2_AS	5'- GCT GTC TGA AAA ATG CTG GT	
mGCK_F	5'- GTA AGG CAC GAA GAC ATA GAC A	60
mGCK_R	5'- TAG TAG CAG GAG ATC ATT GTG G	

Primers for human intestinal organoids:

Pdx1-F	same as above (mouse transgene)	60
Pdx1-R	same as above (mouse transgene)	
hMafA-F	5'- GAG AGC GAG AAG TGC CAA CT	60
hMafA-R	5'- TTC TCC TTG TAC AGG TCC CG	
Ngn3-F	same as above (mouse transgene)	60
Ngn3-R	same as above (mouse transgene)	
hINS-F	5'- GAA CCA ACA CCT GTG CGG CTC A	60
hINS-R	5'- TGC CTG CGG GCT GCG TCT AGT	
hSur1-F	5'- AAG GAG ATG ACC AGC CTC AG	60
hSur1-R	5'- GCC CAC GAA AGT TAT GAG GA	
hKir6.2-F	5'- TGC CTT CCT TTT CTC CAT TG	60
hKir6.2-R	5'- TTC TGC ACG ATG AGG ATC AG	
hSLC2A2-F	5'- TGT GCC ACA CTC ACA CAA GA	60
hSLC2A2-R	5'- GAC AGT GAA AAC CAG GGT CC	
hNkx6.1-F	5'- CTC GTT TGG CCT ATT CGT TG	60
hNkx6.1-R	5'- TCT GTC TCC GAG TCC TGC TT	
hUCN3-F	5'- GGA GGG AAG TCC ACT CTC G	60
hUCN3-R	5'- GAT GGG CTT GGC TTT GTA GA	
hCdx2-F	5'- GGG CTC TCT GAG AGG CAG GT	60
hCdx2-R	5'- GGT GAC GGT GGG GTT TAG CA	
hVillin-F	5'- CCA AAG GCC TGA GTG AAA TC	60
hVillin-R	5'- CCT GGA GCA GCT AGT GAA CA	
hMuc2-F	5'- TGT AGG CAT CGC TCT TCT CA	60
hMuc2-R	5'- GAC ACC ATC TAC CTC ACC CG	

Table S2. List of primary antibodies used in IF staining and western blot analysis (related to Experimental Procedures)

Primary Antibodies:

Antigen	Host	Dilution	Source	Catalogue	Application
Insulin	Guinea Pig	1:100	DAKO	A0564	IF
Glucagon	Rabbit	1:100	DAKO	A0565	IF
E-cadherin	Rat	1:1500	Invitrogen	13-1900	IF
Ngn3	Goat	1:500	G. Gu, Vanderbilt	Gift	IF
Ngn3	Mouse	1:250	DSHB	F25A1B3	IF
Pdx-1	Goat	1:1000	Abcam	ab47383	IF
MafA	Rabbit	1:100	Bethyl Laboratories	IHC-00352	IF
GFP	Chicken	1:1500	Abcam	ab13970	IF
ChroA	Rabbit	1:2000	ImmunoStar	20085	IF
CK-19	Rabbit	1:1000	generated as described previously (Zong et al., 2009)		IF
Ki-67	Rat	1:200	DAKO	M7249	IF
C-peptide	Rabbit	1:400	Cell Signaling	#4593	IF
PH3(Ser10)	Rabbit	1:1000	Cell Signaling	#3377	IF
Ngn3	Goat	1:200	Santa Cruz	SC-13793	WB
Ngn3	Rabbit	1:1000	BCBC	AB2011 (2369B)	WB
Pdx-1	Goat	1:10000	Abcam	ab47383	WB

Supplemental Figures and legends

Figure S1, related to Figure 1

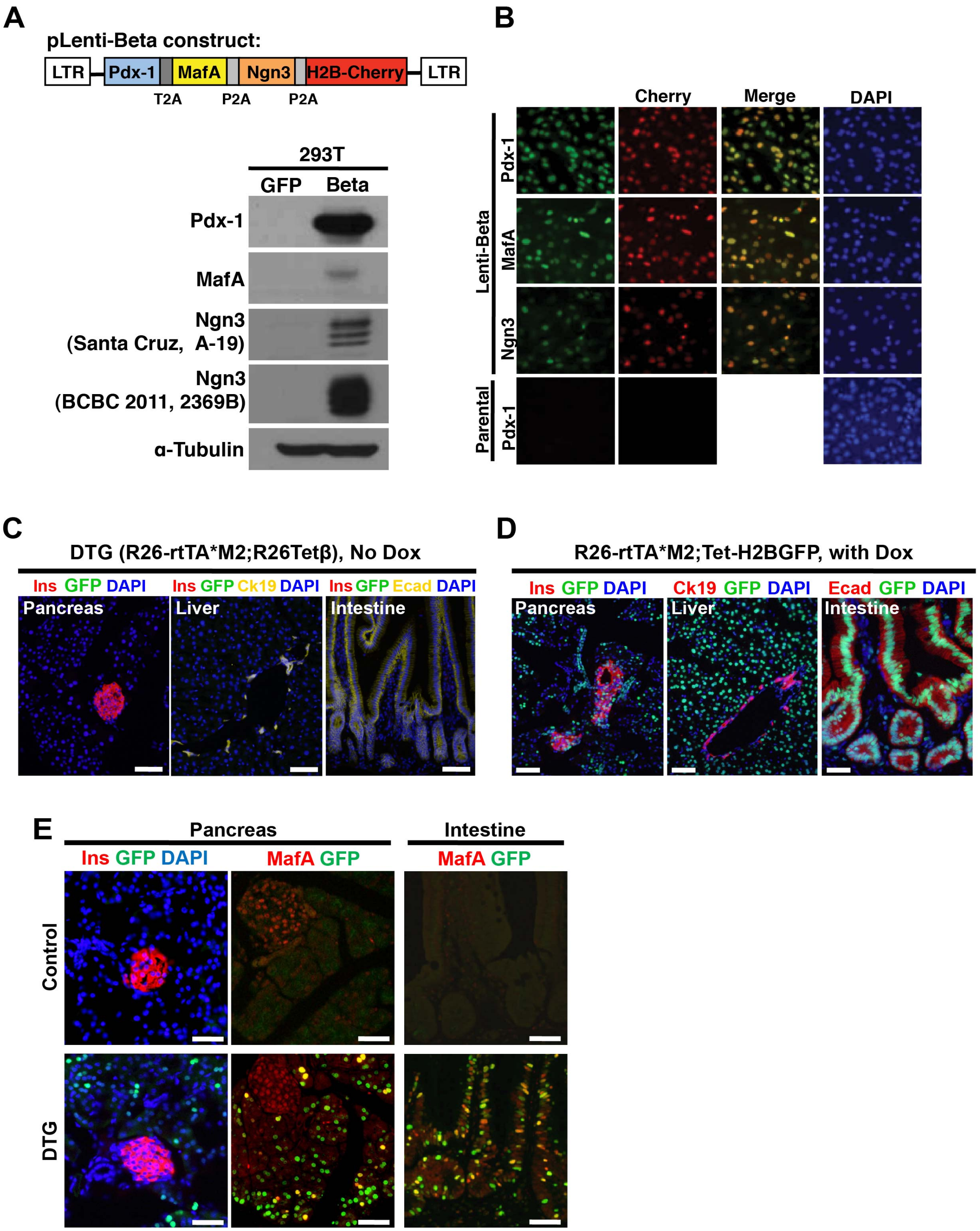

Figure S1. Validation of Tet-Beta polycistronic system both *in vitro* and *in vivo* (related to Figure 1)

(A) A polycistronic vector for expression of Pdx1, MafA, Ngn3, and H2B-Cherry. Top: Schematic representation of the pLenti-Beta construct, showing Pdx-1, MafA, Ngn3, and H2B-Cherry cDNAs linked by 2A peptide sequences (T2A, P2A) in the FUW lentivector backbone. Bottom: Western blot showing detection of Pdx-1, MafA, and Ngn3 proteins following transient transfection of 293T cells with either pLenti-Beta or pLenti-GFP plasmids. Whole cell lysates were extracted 48h after transfection. Bands were detected at the predicted sizes, indicating successful cleavage of each peptide.

(B) Detection of Pdx-1, MafA, Ngn3 and H2B-Cherry proteins by immunofluorescence following infection of a human hepatocyte (HHL) cell line with Lenti-Beta virus.

(C) Representative immunofluorescence staining of tissues from untreated DTG mice stained for GFP, insulin, Ck19, and E-cadherin. Scale bar: 20um

(D) Representative immunofluorescence staining of tissues from Dox-treated *R26-rtTA;Tet-H2BGFP* mice stained for insulin, Ck19, E-cadherin and DAPI, shown with GFP (epifluorescence). Scale bar: 20um

(E) Left: Representative immunofluorescence of pancreata from D4 DTG or control mice stained for insulin and MafA, shown with GFP (epifluorescence). Right: Representative immunofluorescence of intestines from D4 DTG or control stained for MafA, shown with GFP (epifluorescence). Comparable images showing co-expression of Pdx1 and GFP in the pancreas and intestine are shown in Figure 1D and 1E. Scale bar: 25um

Figure S2, related to Figure 2

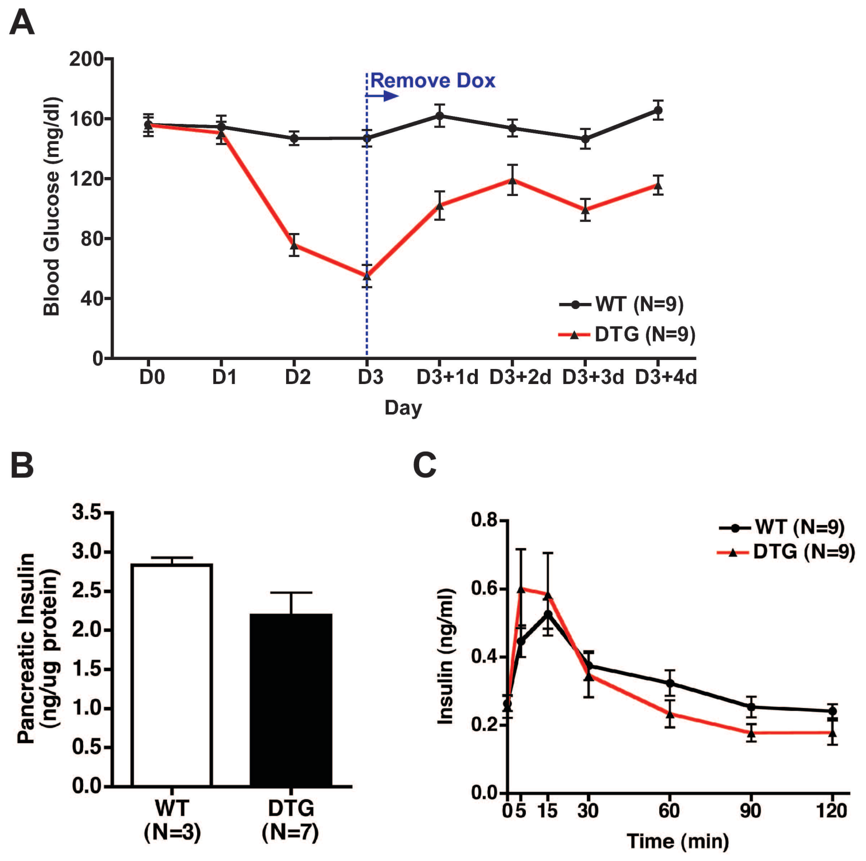

Figure S2. Glucose and insulin levels in DTG animals (related to Figure 2)

(A) Fed blood glucose (BG) levels of control and DTG mice during Dox treatment. DTG animals exhibited a decrease in BG levels on D2 of treatment, and severe hypoglycemia on D3. BG levels rose into the normal range within 1-2d of Dox withdrawal, although they remained lower than BG levels of controls (in contrast to fasting BG, which was the same in both DTG and control animals; see Figure 2D). Data are presented as mean ± S.E.M.

(B) Total pancreatic insulin content measured by ELISA in pancreata from control and DTG animals following 5d de-induction (D3+5d) DTG. Data are presented as mean ± S.E.M.

(C) Serum insulin levels of control and de-induced (D3+5d) DTG animals following an intraperitoneal injection of glucose (IP-GTT). DTG animals show a trend towards an early spike in serum insulin after glucose challenge and lower serum insulin levels after becoming normoglycemic. Data are presented as mean ± S.E.M.

Figure S3, related to Figure 3

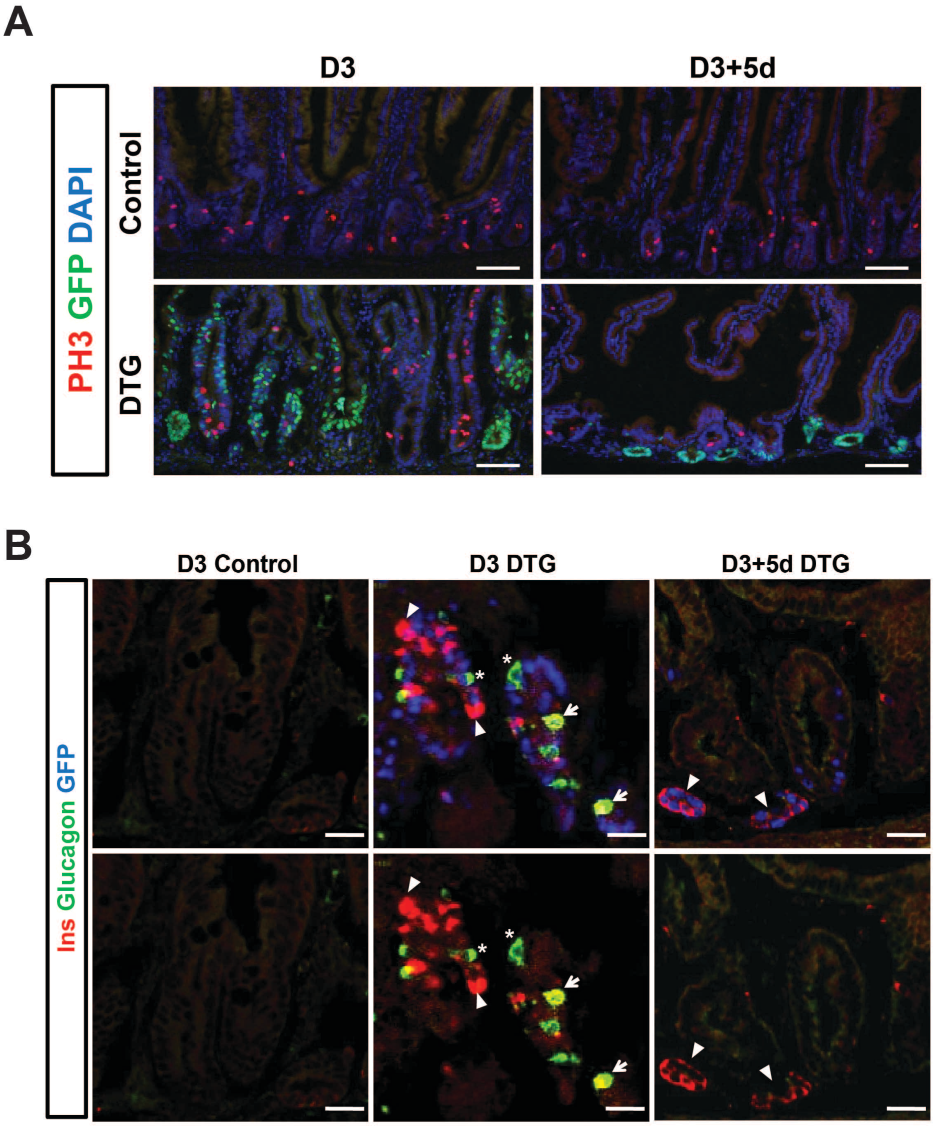

Figure S3. Proliferation and glucagon expression in intestinal GFP$^+$ cells (related to Figure 3)

(A) Representative intestine tissues from D3 and D3+5d animals stained with phospho-histone3 (PH3), GFP and DAPI. Scale bar: 50um.

(B) Immunofluorescence for insulin, glucagon, and GFP in D3 and D3+5d DTG animals. Glucagon$^+$ cells are detected at D3 but not D3+5d. Arrowheads denote cells that are insulin$^+$, asterisks denote cells that are glucagon$^+$, and arrows denote cells that are double positive (insulin$^+$/glucagon$^+$). No glucagon$^+$ or double positive cells were seen in D3+5d neo-islets. Scale bar: 25um.

Figure S4, related to Figure 4

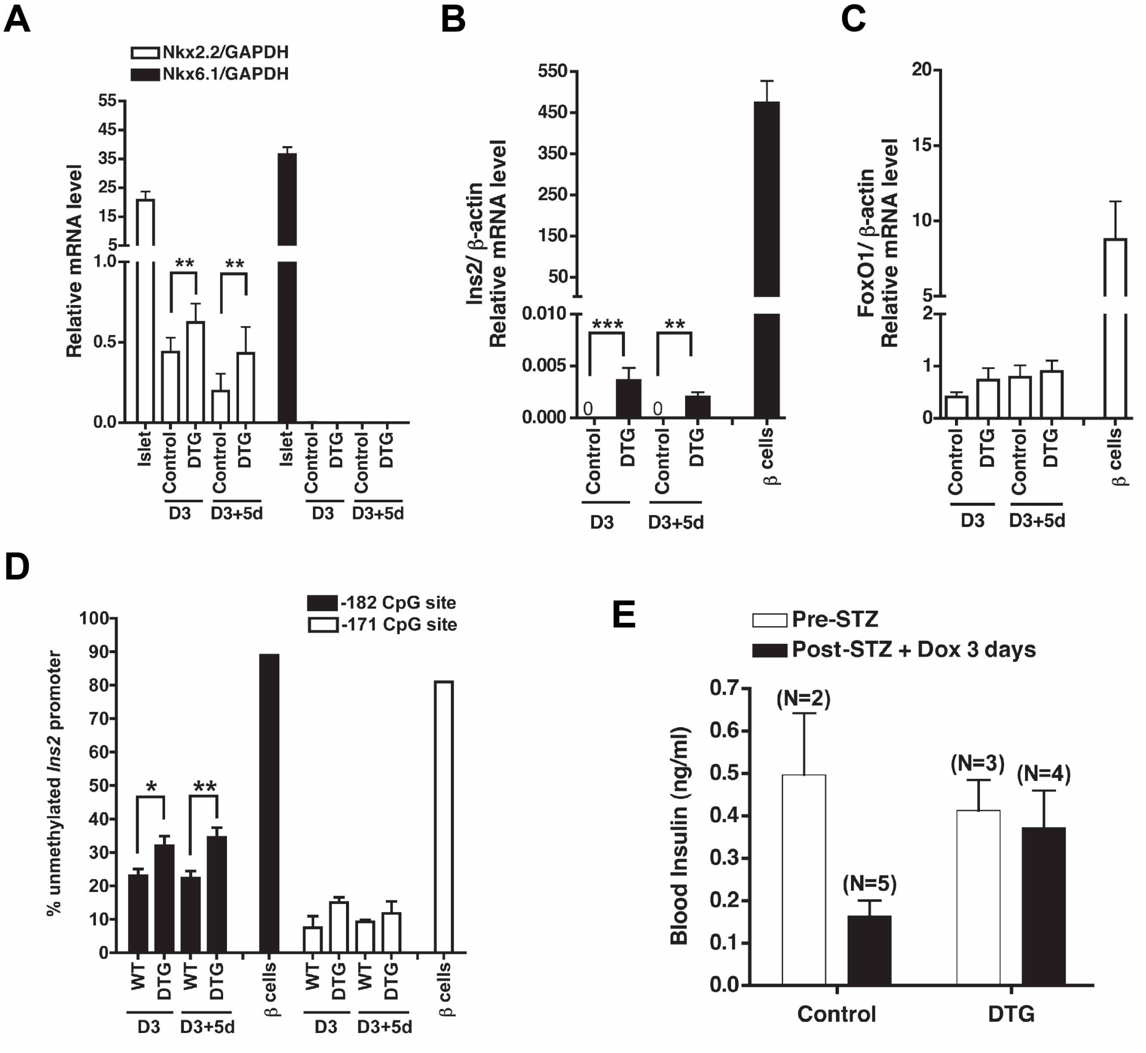

Figure S4. Molecular signatures in GFP$^+$ intestinal crypt cells and serum insulin levels in STZ-treated animals (related to Figure 4D)

(A) Quantitative PCR analysis of *Nkx2.2* and *Nkx6.1* transcripts in isolated crypt cells from D3 or D3+5d DTG animals or littermates. Islets were isolated from wild-type 4-6-wk-old mice. **P<0.01, Student's t-test. Data are presented as mean ± SD. Experiments were performed with two biological replicates and repeated twice. Each biological replicate contains cells isolated from 1~2 mice.

(B and C) Quantitative PCR analysis of *Ins2* and *FoxO1* transcripts in sorted GFP$^+$ crypt cells. Mice were administrated with Dox water for 3 days (D3) follow by regular water for 5 days (D3+5d). Sorted crypt cells were collected at indicated time points. Control

GFP$^+$ cells were sorted from *R26rtTA*M2;Tet-H2BGFP* mice. DTG GFP$^+$ cells were sorted from *R26rtTA*M2;Tetβ* mice. GFP$^+$ β cells were sorted from *MIP-TF* mice (Yong et al., 2011). Significantly elevated level of Ins2 (B) but not FoxO1 (C) transcripts were detected in sorted GFP$^+$ DTG crypt cells compared to control groups. **P<0.01, ***P<0.001, Student's t-test. Data are presented as mean ± SD. Experiments were performed with four biological replicates and repeated twice. Each biological replicate contains sorted cells from 1~2 mice.

(D) Percentage of unmethylated CpG sites on the mouse *Ins2* promoter. Wild-type GFP$^+$ cells were sorted from *Villin-rtTA*M2^{V5};Tet-H2BGFP* mice. DTG GFP$^+$ cells were sorted from *R26rtTA*M2;Tetβ* mice. GFP$^+$ β cells were sorted from *MIP-TF* mice. Sorted crypt cells were collected at indicated time points followed by pyrosequencing analysis at two CpG sites (-171 and -182, (Kuroda et al., 2009)). *P<0.05, **P<0.01, Student's t-test. Data are presented as mean ± S.E.M. Experiments were performed with two biological replicates. Each biological replicate contains sorted cells from 1~2 mice.

(E) Serum was prepared from control or DTG animals prior to STZ treatment or after STZ–Dox treatment (4 days after STZ injection followed by 3 days of Dox treatment). Insulin concentration was measured by ELISA after a 1h fast.

Figure S5, related to Figure 6

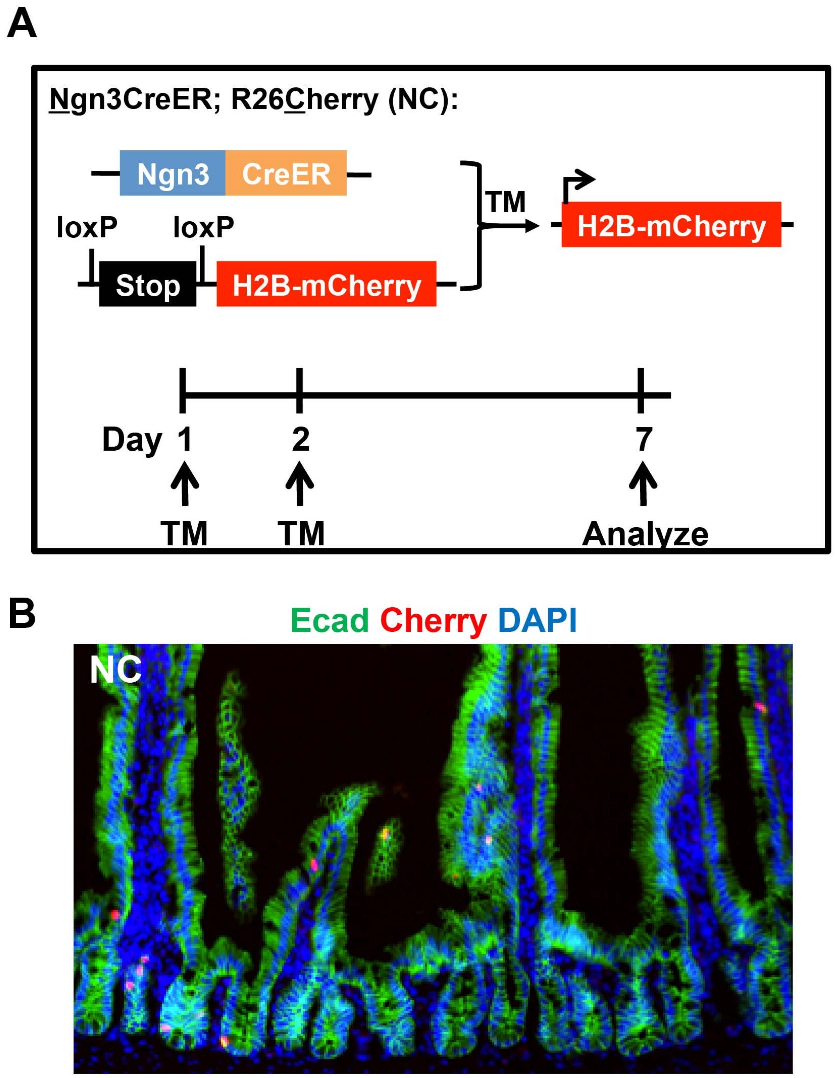

Figure S5. Ngn3-lineage labeling in the intestine (related to Figure 6)

(A) Schematic representation of experimental design. *Ngn3CreER; R26Cherry* (NC) mice were given 8mg tamoxifen (TM) by intraperitoneal injection on two consecutive days. On day 7 (6 days after the first injection), intestines were harvested and analyzed for H2BCherry epifluorescence.

(B) Representative image of intestinal tissue stained for E-cadherin (Ecad) and DAPI, shown with H2BCherry epifluorescence.

Figure S6, related to Figure 7

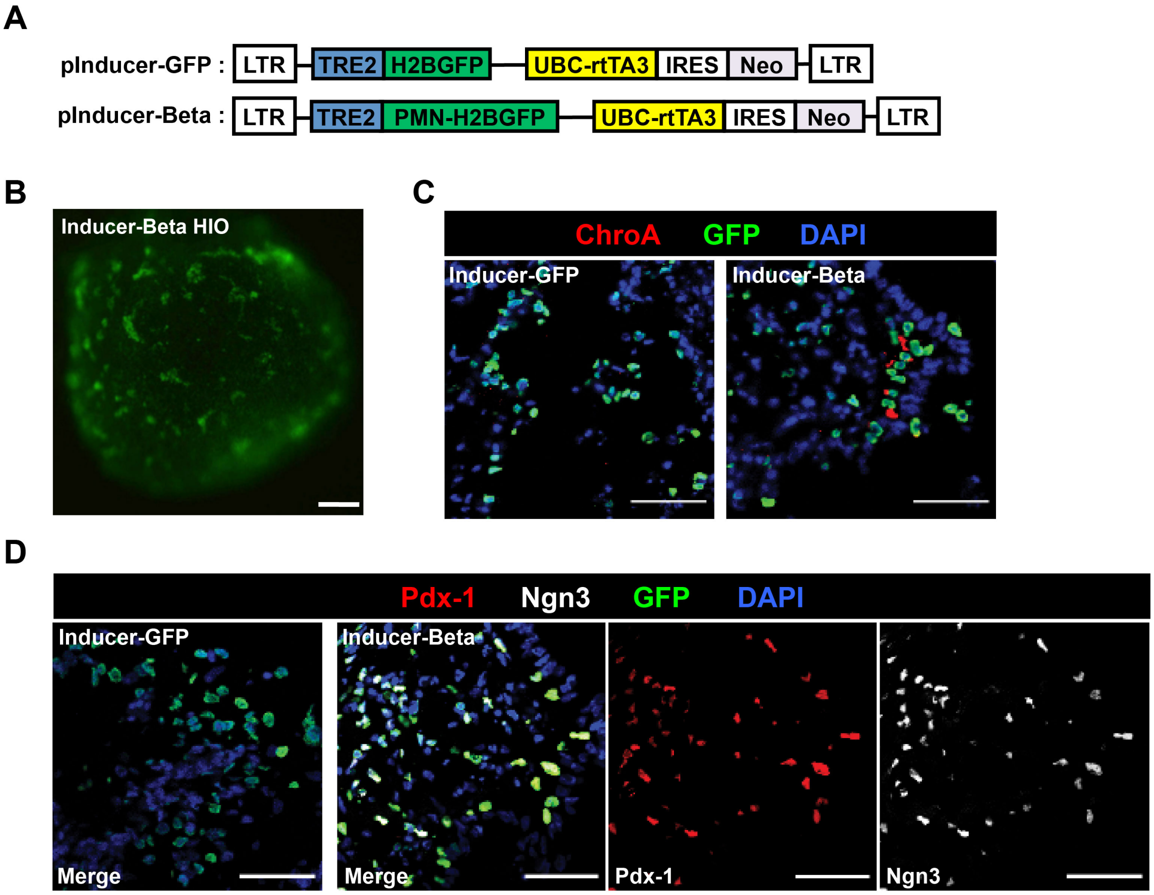

Figure S6. Generation of human intestinal organoids with inducible PMN factors (related to Figure 7)

(A) Inducible expression of PMN factors by a single lenti-vector. Schematic representation of the pInducer-GFP and pInducer-Beta lenti-vectors. Human ES cells were infected with pInducer-GFP or pInducer-Beta virus to generate stable cell lines. Detailed methods are provided in Extended Experimental Procedures.

(B) GFP^+ clusters were seen in human intestinal organoids (HIOs) carrying the Inducer-Beta viral insert after 10d Dox treatment.

(C-D) Inducer-Beta-derived HIOs were treated with Dox for 10d and cryosections were examined by co-immunofluorescence for GFP and ChroA (C) or GFP, Pdx1 and Ngn3 (D). Scale bars: 200um (B); 50um (C-D).

Supplemental References:

Cui, W., Taub, D.D., and Gardner, K. (2007). qPrimerDepot: a primer database for quantitative real time PCR. Nucleic Acids Res *35*, D805-809.

Kuroda, A., Rauch, T.A., Todorov, I., Ku, H.T., Al-Abdullah, I.H., Kandeel, F., Mullen, Y., Pfeifer, G.P., and Ferreri, K. (2009). Insulin gene expression is regulated by DNA methylation. PLoS One *4*, e6953.

Madison, B.B., Dunbar, L., Qiao, X.T., Braunstein, K., Braunstein, E., and Gumucio, D.L. (2002). Cis elements of the villin gene control expression in restricted domains of the vertical (crypt) and horizontal (duodenum, cecum) axes of the intestine. J Biol Chem *277*, 33275-33283.

McCracken, K.W., Howell, J.C., Wells, J.M., and Spence, J.R. (2011). Generating human intestinal tissue from pluripotent stem cells in vitro. Nat Protoc *6*, 1920-1928.

Meerbrey, K.L., Hu, G., Kessler, J.D., Roarty, K., Li, M.Z., Fang, J.E., Herschkowitz, J.I., Burrows, A.E., Ciccia, A., Sun, T.*, et al.* (2011). The pINDUCER lentiviral toolkit for inducible RNA interference in vitro and in vivo. Proc Natl Acad Sci U S A *108*, 3665-3670.

Sato, T., Vries, R.G., Snippert, H.J., van de Wetering, M., Barker, N., Stange, D.E., van Es, J.H., Abo, A., Kujala, P., Peters, P.J.*, et al.* (2009). Single Lgr5 stem cells build crypt-villus structures in vitro without a mesenchymal niche. Nature *459*, 262-265.

Spence, J.R., Mayhew, C.N., Rankin, S.A., Kuhar, M.F., Vallance, J.E., Tolle, K., Hoskins, E.E., Kalinichenko, V.V., Wells, S.I., Zorn, A.M.*, et al.* (2011). Directed differentiation of human pluripotent stem cells into intestinal tissue in vitro. Nature *470*, 105-109.

Tumbar, T., Guasch, G., Greco, V., Blanpain, C., Lowry, W.E., Rendl, M., and Fuchs, E. (2004). Defining the epithelial stem cell niche in skin. Science *303*, 359-363.

Yong, J., Rasooly, J., Dang, H., Lu, Y., Middleton, B., Zhang, Z., Hon, L., Namavari, M., Stout, D.B., Atkinson, M.A.*, et al.* (2011). Multimodality imaging of beta-cells in mouse models of type 1 and 2 diabetes. Diabetes *60*, 1383-1392.

Zong, Y., Panikkar, A., Xu, J., Antoniou, A., Raynaud, P., Lemaigre, F., and Stanger, B.Z. (2009). Notch signaling controls liver development by regulating biliary differentiation. Development *136*, 1727-1739.
